# Examining the interaction between prenatal stress and polygenic risk for attention-deficit/hyperactivity disorder on brain growth in childhood: Findings from the DREAM BIG consortium

**DOI:** 10.1002/dev.22481

**Published:** 2024-05

**Authors:** Mónica López-Vicente, Eszter Szekely, Marie-Elyse Lafaille-Magnan, J. Bruce Morton, Tim F. Oberlander, Celia M. T. Greenwood, Ryan L. Muetzel, Henning Tiemeier, Anqi Qiu, Ashley Wazana, Tonya White

**Affiliations:** 1 Department of Child and Adolescent Psychiatry and Psychology, Erasmus MC University Medical Center, Rotterdam, The Netherlands; 2 The Generation R Study Group, Erasmus MC University Medical Center, Rotterdam, The Netherlands; 3 McGill University – Faculty of Medicine and Health Sciences, Montréal, Québec, Canada; 4 Lady Davis Institute for Medical Research, Jewish General Hospital, Montréal, Québec, Canada; 5 Department of Psychology, The University of Western Ontario, London, Ontario, Canada; 6 School of Population and Public Health, Faculty of Medicine, University of British Columbia, Vancouver, Canada; 7 Department of Pediatrics, Faculty of Medicine, University of British Columbia, Vancouver, Canada; 8 Department of Human Genetics, McGill University, Montréal, Québec, Canada; 9 Department of Epidemiology, Biostatistics and Occupational Health, McGill University, Montréal, Québec, Canada; 10 Gerald Bronfman Department of Oncology, McGill University, Montréal, Québec, Canada; 11 Department of Radiology and Nuclear Medicine, Erasmus MC University Medical Center, Rotterdam, The Netherlands; 12 Department of Social and Behavioral Science, Harvard T. H. Chan School of Public Health, Boston, Massachusetts, USA; 13 Department of Biomedical Engineering, National University of Singapore, Singapore, Singapore; 14 NUS (Suzhou) Research Institute, National University of Singapore, Suzhou, China; 15 The N.1 Institute for Health, National University of Singapore, Singapore, Singapore; 16 Institute of Data Science, National University of Singapore, Singapore, Singapore; 17 Department of Biomedical Engineering, the Johns Hopkins University, Baltimore, Maryland, USA; 18 Section on Social and Cognitive Developmental Neuroscience, National Institute of Mental Health, Bethesda, Maryland, USA

**Keywords:** attention-deficit/hyperactivity disorder, birth cohort, longitudinal design, polygenic score, prenatal stress, structural magnetic resonance imaging

## Abstract

This study explored the interactions among prenatal stress, child sex, and polygenic risk scores (PGS) for attention-deficit/hyperactivity disorder (ADHD) on structural developmental changes of brain regions implicated in ADHD. We used data from two population-based birth cohorts: Growing Up in Singapore Towards healthy Outcomes (GUSTO) from Singapore (*n* = 113) and Generation R from Rotterdam, the Netherlands (*n* = 433). Prenatal stress was assessed using questionnaires. We obtained latent constructs of prenatal adversity and prenatal mood problems using confirmatory factor analyses. The participants were genotyped using genome-wide single nucleotide polymorphism arrays, and ADHD PGSs were computed. Magnetic resonance imaging scans were acquired at 4.5 and 6 years (GUSTO), and at 10 and 14 years (Generation R). We estimated the age-related rate of change for brain outcomes related to ADHD and performed (1) prenatal stress by sex interaction models, (2) prenatal stress by ADHD PGS interaction models, and (3) 3-way interaction models, including prenatal stress, sex, and ADHD PGS. We observed an interaction between prenatal stress and ADHD PGS on mean cortical thickness annual rate of change in Generation R (i.e., in individuals with higher ADHD PGS, higher prenatal stress was associated with a lower rate of cortical thinning, whereas in individuals with lower ADHD PGS, higher prenatal stress was associated with a higher rate of cortical thinning). None of the other tested interactions were statistically significant. Higher prenatal stress may promote a slower brain developmental rate during adolescence in individuals with higher ADHD genetic vulnerability, whereas it may promote a faster brain developmental rate in individuals with lower ADHD genetic vulnerability.

## 1 | INTRODUCTION

Attention-deficit/hyperactivity disorder (ADHD) is one of the most common neurodevelopmental disorders during childhood, affecting approximately 5% of individuals worldwide (Polanczyk et al., 2007). The heritability of ADHD has been estimated to be 60%–80% (Lichtenstein et al., 2010). Like other psychiatric disorders, ADHD has a polygenic architecture, with multiple common genetic variants of small effect contributing to its etiology (Demontis et al., 2019). Polygenic risk scores (PGSs), calculated as a weighted sum of trait-associated alleles, are commonly used to estimate the individual polygenic vulnerability of complex phenotypes (Choi et al., 2020).

Prenatal stress, such as socioeconomic adversity and maternal mental disorders during pregnancy, can increase a child’s likelihood of developing ADHD, both independently (Linnet et al., 2003) and in combination with genetic risk (Banerjee et al., 2007; Nigg et al., 2010). An important limitation of the gene-environment interaction studies in ADHD, however, is that they have mostly examined the interplay between single nucleotide polymorphisms (SNPs) and single environments. Given the polygenic architecture of ADHD and the multiple prenatal factors that influence this disorder, a more comprehensive approach is needed to fully capture the complex interplay of genetic and environmental factors in ADHD and its underlying neurobiology.

ADHD involves structural alterations, such as basal ganglia and cerebellar volume reductions (Alemany et al., 2019; Frodl & Skokauskas, 2012; Hoogman et al., 2017; Mous et al., 2015; Qiu et al., 2009), less cortical thickness (Hoogman et al., 2019; Mous et al., 2017; Qiu et al., 2012), and smaller cortical surface area (Hoogman et al., 2019). Studying neural correlates of psychopathology is important for mapping out the influence of genetic and prenatal environmental factors on ADHD symptoms, given that they reflect “endophenotypes” that lie closer to the underlying etiology (Gottesman & Gould, 2003; Insel & Cuthbert, 2009). The developmental course of a complex trait is particularly sensitive to the interaction of multiple genetic and environmental factors over time (Giedd et al., 2008). However, the incorporation of repeated brain measures in gene-environment interaction studies is rare. On the contrary, there is extensive research about the development of brain measures in relation to ADHD. In general, children with ADHD show delayed peak values of development in the cortical thickness and surface area, particularly in the right lateral prefrontal cortex (Shaw et al., 2007, 2011, 2012). This structure has consistently been associated with ADHD (Faraone et al., 2015; Giedd et al., 2001), and it is involved in cognitive functions like executive control, working memory, and motivation (Casey et al., 2002; Rogers et al., 1999).

Sex differences have also been reported both at the level of brain and behavior (Gur & Gur, 2017). Although the trajectories of brain development in children with ADHD have not yet been studied separately for boys and girls, it is known that total cerebral volume peaks earlier in females (10.5 years) than males (14.5 years) (Lenroot et al., 2007). Furthermore, the impact of prenatal stress on learning and memory has been observed to be larger in males than in females, whereas females appear more susceptible to anxiety and depression (Glover & Hill, 2012; Graham et al., 2019) and to limbic circuitry alterations (Soe et al., 2018).

In this study, we aimed to examine: (1) the interaction between prenatal stress (socioeconomic adversity and maternal mood problems during pregnancy) and sex on annual rates of change of brain regions implicated in ADHD and (2) prenatal stress interaction with ADHD PGS on the rates of change in these brain regions. Additionally, we explored 3-way interaction models that included prenatal stress, child’s sex, and ADHD PGS. We used data from two cohorts with available magnetic resonance imaging (MRI) data at two time points spanning different age periods: Growing Up in Singapore Towards healthy Outcomes (GUSTO) from 4 to 6 years and the Generation R Study from 10 to 14 years. These age periods comprise key stages of brain development, particularly for the prefrontal cortex (Bethlehem et al., 2022; Gogtay et al., 2004).

## 2 | METHODS

### 2.1 | Participants

This study is part of the Developmental Research in Environmental Adversity, Mental Health, BIological susceptibility, and Gender (DREAM-BIG) project, a multicenter consortium of population-based prenatal cohorts (Neumann et al., 2022; Szekely et al., 2021). We used data from two cohorts: the GUSTO study, from Singapore, and the Generation R Study, from Rotterdam (the Netherlands). The GUSTO study recruited 1,247 pregnant women between June 2009 and September 2010. The participants were of Chinese, Malay, or Indian ethnicity. MRI data were collected at a mean age of 4.5 and 6 years (Wen et al., 2017). A total of 113 participants had genetic, prenatal stress, and MRI data (Figure S1). Generation R recruited 9,778 pregnant women between April 2002 and January 2006. For this study, we only included the participants with European ancestry (Medina-Gomez et al., 2015). MRI data were collected at a mean age of 10 and 14 years (White et al., 2018). A total of 433 participants had genetic, prenatal stress, and MRI data (Figure S2).

The GUSTO study was approved by the National Healthcare Group Domain Specific Review Board and the SingHealth Centralized Institutional Review Board. Written informed consent was obtained from mothers. The Generation R Study was approved by the local medical ethics committee of the Erasmus University Medical Center. All parents provided written informed consent and children provided assent (younger than 12 years) or consent (12 years or older).

### 2.2 | Measurements

#### 2.2.1 | Predictors

Child sex and date of birth were determined from medical records obtained at birth.

##### 2.2.1.1 Prenatal stress

To obtain prenatal adversity scores (A-factor), we performed hierarchical second-order confirmatory factor analysis (CFA) in GUSTO and Generation R separately using the lavaan R package (Rosseel, 2012). The A-factor score is based on the previously calculated cumulative environmental risk score (Rijlaarsdam et al., 2016). We included items of stressful life events (i.e., death of a relative), contextual risks (i.e., financial problems), personal/family risks (i.e., low education level), and interpersonal risks (i.e., low social support). The exact items and administration times are described in the Appendix (S1 and S2). Prenatal mood problems scores (M-factor) were constructed previously in Generation R using a CFA assuming a bifactor structure (Szekely et al., 2021) in the lavaan R package (Rosseel, 2012). This measure was also replicated in two other prenatal cohorts, the Avon Longitudinal Study of Parents and Children (ALSPAC) and the Maternal Adversity, Vulnerability, and Neurodevelopment (MAVAN) (Szekely et al., 2021). We used the same method to compute the M-factor in GUSTO. The exact items used are detailed in the Appendix (S3 and S4). CFA details and model fit indices are described in Appendix S5 and Table S1. Generally, model fit indices were acceptable. Factor scores were extracted and standardized before analyses. Higher scores represent higher stress.

##### 2.2.1.2 Genotyping and polygenic risk scoring

The genetic data quality assessment procedure and imputation are described in Appendix S6. ADHD PGSs were computed using publicly available ADHD genome-wide association study (GWAS) mega-analysis results from the Psychiatric Genomics Consortium (Demontis et al., 2023) using *N* = 38,691 cases and *N* = 186,843 controls. We used the PRSice 2 software to calculate the scores with the following *p* value thresholds: 1, .5, .4, .3, .2, .1, .05, .01, .001, .0001, 1 × 10^−5^, 1 × 10^−6^, 1 × 10^−7^, 5 × 10^−8^, and 1 × 10^−8^. The best PGS threshold for each brain outcome was selected based on cross-validation results (see statistical analyses). Given that psychiatric PGSs have shown low specificity in the prediction of psychopathology (Neumann et al., 2022), we used two other PGSs, major depression (Howard et al., 2019) and schizophrenia (Schizophrenia Working Group of the Psychiatric Genomics Consortium, 2014), to test the specificity of our findings. In GUSTO, we only used the major depression PGS, given the association found between schizophrenia PGS and ancestry (Curtis, 2018). The brain structural correlates of major depression and schizophrenia are different from the alterations related to ADHD, although the PGSs for major depression and ADHD were expected to be slightly correlated (Neumann et al., 2022). In addition, these PGSs are comparable in terms of their associations with general psychopathology in Generation R (Neumann et al., 2022). Threshold selection was performed the same way as for the ADHD PGS.

The population structure of both cohorts was evaluated using principal component analysis. In GUSTO, the first three principal components from the GWAS analysis were the most informative of genetic ancestry (De Lima et al., 2020). In Generation R, these components were computed using European participants only, and the first four principal components from the GWAS analysis were the most informative (Medina-Gomez et al., 2015).

#### 2.2.2 | Outcomes

MRI acquisition details and quality assessment procedures are described in Appendix S7. Images were processed using FreeSurfer (Fischl, 2012). The standard reconstruction was conducted, and surface-based models of white matter and gray matter were generated. Subcortical structures were automatically labeled, and volumes in cubic millimeter were extracted. Cortical thickness was estimated at each point (vertex) along the cortical ribbon, and each point was also automatically assigned an anatomical label according to a predefined atlas (Desikan et al., 2006).

Based on prior ADHD literature (Faraone et al., 2015; Giedd et al., 2001; Hoogman et al., 2017, 2019; Shaw et al., 2011, 2012, 2014), we included the caudate, putamen, and cerebellar volumes, total brain volume, cortical thickness, and surface area of the entire brain and of the right lateral prefrontal cortex (divided in the dorsolateral prefrontal cortex, which includes the superior frontal, rostral middle frontal, and caudal middle frontal gyri, as labeled by FreeSurfer; and the ventrolateral prefrontal cortex, which includes the pars opercularis, pars triangularis, and pars orbitalis). We computed means of left and right hemisphere values. Because the subcortical structures (caudate and putamen) and the cerebellum scale with global brain size, these measures were adjusted for intracranial volume (ICV) by computing ratios. The surface area of two lateral prefrontal cortex regions was adjusted by the total surface area.

#### 2.2.3 | Covariates

Given that the A-factor includes an exhaustive list of social-environmental risk factors, the models were only adjusted for genetic ancestry. In GUSTO, the models were adjusted for the first three genetic principal components (De Lima et al., 2020), and in Generation R, for the first four genetic principal components (Medina-Gomez et al., 2015). Attention problems of the children, used for descriptive purposes only, were measured at the ages of 4 in GUSTO and 10 years old in Generation R using the Child Behavior Checklist (CBCL) Attention problem scale (Achenbach & Rescorla, 2001).

### 2.3 | Statistical analyses

The analyses were performed separately in GUSTO and Generation R using the R Statistical Software (version 3.6.0). In-line with previous studies (Szekely et al., 2018; Vijayakumar et al., 2014), we estimated the intra-individual age-related rate of change in brain outcomes:

$$100\times \left(\frac{\text{outcome}\;\text{visit}\;2\;-\;\text{outcome}\;\text{visit}\;1}{\text{outcome}\;\text{visit}\;1}\right)\times \left(\frac{1}{\text{age}\;\text{visit}\;2\;-\;\text{age}\;\text{visit}\;1}\right)$$

Positive values indicated annual increases in the brain outcome measures, whereas negative values indicated annual decreases in the brain outcome measures. Then, using linear regression, this change rate was adjusted for the age at baseline scan. After adjustment, residualized values were normalized using rank-based inverse normal transformation, Blom’s formula: $\phi (\frac{\text{rank}-\frac{3}{8}}{n+\frac{1}{4}})$.

We fitted interaction models using the Latent Environmental and Genetic InTeraction package in R (Jolicoeur-Martineau et al., 2020). This package allows us to include multiple genetic and environmental variables simultaneously, where the genetic score weights, the environmental score weights, and the main model parameters are estimated in parts, assuming the other parameters to be constant. Two types of models were performed: (1) prenatal stress (A and M factors, which were entered together and separately in the models) by sex interaction models adjusted for genetic ancestry, and (2) prenatal stress (A and M factors, together and separate) by PGS for ADHD adjusted for sex and genetic ancestry. Additionally, we performed 3-way interaction models, including the three variables of interest (prenatal stress, sex, and ADHD PGS), entering the A and M factors together. The dependent variables in these models were the residualized rate of change values corresponding to the 10 brain outcomes. To select the PGS thresholds, we performed 5-fold cross-validations on models that included the stress factor scores and every PGS threshold at a time for each brain outcome. We selected the PGS thresholds that showed the highest *R*^2^.

The results were corrected for multiple testing using the Bonferroni correction adjusting for the effective number of tests (Galwey, 2009). This approach accounts for the nonindependence between variables, in this case, the correlation between the change values of the 10 brain outcomes. This method assumes that the sample size exceeds the number of tests and provides a metric for the effective number of tests. The *p* value threshold adjusted for an effective number of tests was .007 in GUSTO and .006 in Generation R.

To test the specificity of the findings concerning the ADHD PGS, all models that showed significant associations were rerun by replacing the ADHD PGS by the PGS for major depression in GUSTO, and major depression and schizophrenia PGSs in Generation R. We also repeated the main analyses excluding children taking psychostimulant medication for ADHD, in Generation R, where this information was available.

## 3 | RESULTS

### 3.1 | Descriptive analysis

The descriptive Table 1 shows the main characteristics of the participants in each cohort. Girls represented 56.6% of the sample in GUSTO, whereas half of the Generation R participants were girls. The percentage of mothers with higher education was 58.4% and 66% in GUSTO and Generation R, respectively. In Generation R, maternal mood problems during pregnancy were lower in the included sample than in the original sample with missing data (BSI Global Severity Index mean = 0.16; SD = 0.2 vs. mean = 0.32; SD = 0.4). The CBCL attention problems T score was lower in the included sample (mean = 53.44; SD = 4.74) than in the original sample (mean = 54.12; SD = 5.63) and the subgroup of participants with low-quality MRI data (mean = 54.28; SD = 5.38).

The distributions of the ADHD, schizophrenia, and major depression PGSs at optimal thresholds are represented in Figures S3 and S4. We did not observe major differences in the distributions between the selected and the excluded samples. The Spearman correlation between the ADHD and the major depression PGSs in GUSTO was *r* = .15 (*p* value = .267). In Generation R, the correlation between ADHD and schizophrenia PGSs was *r* = .04 (*p* value = .606), between ADHD and major depression was *r* = .12 (*p* value = .078), and between schizophrenia and major depression, *r* = .16 (*p* value < .001). Correlation matrices among all the tested PGSs are depicted in Figures S5 and S6. The A and M factor scores were distributed similarly as in the original sample in both cohorts (Figures S7 and S8). The Spearman correlation between both scores was *r* = .17 (*p* value = .014) in GUSTO and *r* = .40 (*p* value < .001) in Generation R.

### 3.2 | Rate of change in brain outcome measures

Figures S9 and S10 show the brain outcome measures in relation to the age at scan. Between ages 4.5 and 6 (GUSTO), there was a general increasing trend in the caudate, putamen, and cerebellar volumes, adjusted for ICV (Figure S9), whereas between 10 and 14 years (Generation R), there was a decrease in these measures (Figure S10). The cortical thickness decreased over time in both cohorts. In contrast, the surface area and total brain volume increased slightly in both cohorts. The unadjusted brain outcome measures in relation to age are represented in Figures S11 and S12. The distributions of the annual rates of change residualized for the age at baseline scan before normalization are shown in Figures S13 and S14.

In GUSTO, we observed positive correlations between the residualized and normalized change values of the putamen and the caudate (Figure S15). In Generation R, the change values of the caudate, putamen, and cerebellar volumes were positively correlated (Figure S16). In both cohorts, total brain volume was positively correlated with cortical thickness and global surface area.

### 3.3 | The moderation of prenatal stress by sex

The prenatal stress-by-sex interaction models showed no significant interactions on any outcome in either GUSTO or Generation R (Table 2). Similar results were observed when the prenatal stress factors (A and M) were included in separate models (Table S2).

### 3.4 | The moderation of prenatal stress by genetic susceptibility

**FIGURE 1:**
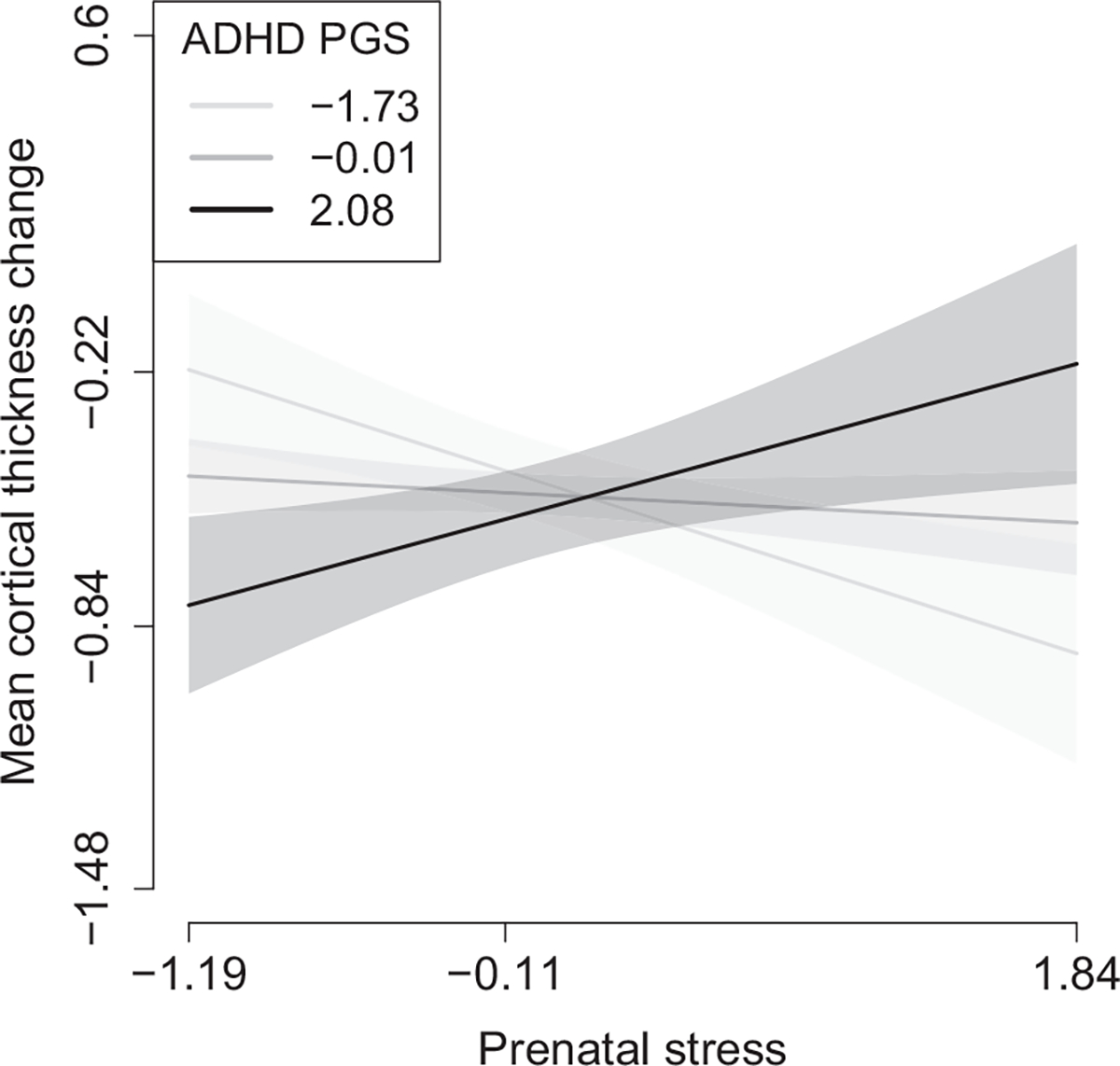
Attention-deficit/hyperactivity disorder (ADHD) polygenic risk (PGS) by prenatal stress (A and M factors) interaction on cortical thickness change^a^ in Generation R. ^a^Annual rates of change residualized on age at baseline scan before normalization. The values depicted in the y-axis indicate the 2.5, 25, 75, and 97.5th percentiles of the outcome. The values depicted in the x-axis and in the legend indicate the 2.5, 50, and 97.5th percentiles of the prenatal stress score and the PGS, respectively.

In GUSTO, we observed a statistically significant (*p* < .05) interaction between prenatal stress and ADHD PGS on the right dorsolateral prefrontal cortex (DLPFC) cortical thickness change, which was driven by the A factor (Tables 3 and S3); however, it did not remain significant after multiple testing correction. Prenatal stress was related positively to DLPFC cortical thickness change (less thinning) at higher ADHD PGS and negatively (greater thinning) at lower ADHD PGS. In Generation R, we found an interaction between prenatal stress (A factor) and the ADHD PGS on mean cortical thickness change that was significant after multiple testing correction (*p* = .0004) (Table 3). Similar to the interaction observed in GUSTO, prenatal stress was related positively to mean cortical thickness growth (less thinning) at higher ADHD PGS and negatively (greater thinning) at lower ADHD PGS. At lower levels of stress, individuals with higher ADHD PGS showed greater cortical thinning than individuals with lower ADHD PGS, whereas, at higher stress levels, individuals with a higher ADHD PGS showed less thinning than individuals with lower ADHD PGS (Figure 1). Similar results were observed when only the A factor was included in the models (Table S3). Other interactions were observed, namely, on the caudate volume, the DLPFC and ventrolateral prefrontal cortex (VLPFC) cortical thickness, and the VLPFC surface area, but they did not remain after multiple testing correction. In GUSTO, no interactions were observed using the major depression PGS (Table S4). The interaction found in Generation R was also observed, although attenuated, when we used the major depression PGS instead of ADHD PGS in the model (Table S4). In contrast, the interaction on the caudate volume change was stronger when both alternative PGS were used instead of ADHD PGS. Similar effect estimates were seen after excluding children taking ADHD medication in Generation R (*n* = 17) (Table S5).

### 3.5 | The moderation of prenatal stress by sex and genetic susceptibility

The 3-way interaction models showed no significant findings after multiple testing corrections (Table S6). However, some results are worth noting as the *p* values were < .05 before the correction. In girls from GUSTO, we observed positive relationships between prenatal stress and growth both in the cerebellum and in total brain volume at higher ADHD PGS. The associations were negative at lower ADHD PGS. However, we observed the opposite in the caudate and the surface area. At higher ADHD PGS, prenatal stress was negatively associated with growth, whereas at lower ADHD PGS, the associations were positive. In girls from Generation R, prenatal stress was positively associated with surface area growth at higher ADHD PGS, whereas it was negatively associated at lower ADHD PGS.

## 4 | DISCUSSION

This study explored the interactions between prenatal stress factor scores, child sex, and the PGS for ADHD, on developmental changes in brain regions implicated in ADHD. We found an interaction between prenatal stress (mainly driven by prenatal adversity) and ADHD PGS on mean cortical thickness annual rate of change between 10 and 14 years of age. Higher prenatal stress was associated with less cortical thinning at higher ADHD PGS, and it was associated with a greater cortical thinning at lower ADHD PGS. At lower prenatal stress levels, individuals with higher ADHD PGS showed a greater cortical thinning than individuals with lower ADHD PGS. At higher prenatal stress levels, individuals with higher ADHD PGS showed less cortical thinning, as compared to individuals with lower ADHD PGS. None of the other tested interactions were significant after multiple testing corrections.

The brain developmental patterns observed during both time periods are consistent with previous literature (Bethlehem et al., 2022; Teffer & Semendeferi, 2012). The annual rate of change values was larger in GUSTO than in Generation R, as expected due to the younger age. The caudate, putamen, and cerebellum changed in the expected directions (Bethlehem et al., 2022; Shaw et al., 2014). Regarding cortical thickness, most research reports very early developmental peaks, around 1 and 5 years old, which is consistent with our results (Bethlehem et al., 2022; Gilmore et al., 2018). The very small change observed in total surface area and total brain volume, particularly in Generation R, is consistent with previous studies, which reported peak values around 8 and 12 years old (Gilmore et al., 2018; Shaw et al., 2012).

We did not observe interactions between prenatal stress and child sex on the development of brain regions implicated in ADHD. A recent study in Generation R reported no associations between prenatal adversity and brain morphology at 10 years old (Hidalgo et al., 2022). The high socioeconomic status of the participants could have influenced these results. It is possible that we were not able to detect differences on the associations between prenatal stress and the brain development by sex because the prenatal stress levels were too low in our participants. Another explanation could be that differences on the impact of prenatal stress on the brain between boys and girls are observed earlier in development and catches up at a later stage. In any case, if more subtle interaction effects are present, larger sample sizes would be required for detection.

In Generation R, prenatal stress (mainly prenatal adversity, which included stressful life events, contextual, personal/family, and interpersonal risks) was differently related to mean cortical thickness annual rate of change, depending on the genetic vulnerability for ADHD. In individuals with higher ADHD PGS, higher prenatal stress led to less cortical thinning. In individuals with lower ADHD PGS, higher prenatal stress led to a greater cortical thinning. This finding suggests that ADHD PGS moderates the impact that prenatal stress has on the brain developmental rate during adolescence. Higher prenatal stress levels may promote a slower brain developmental rate during the studied period (10–14 years) in individuals with higher PGS, whereas these higher levels of stress may promote a faster developmental rate in individuals with lower PGS. The fact that at higher prenatal stress levels, individuals with higher ADHD PGS showed less cortical thinning, as compared to individuals with lower ADHD PGS was consistent with the previous research (Shaw et al., 2007, 2011). These studies found delays in cortical thinning in children with ADHD symptoms, and the developmental rate was lower compared to children without ADHD symptoms. The greater cortical thinning observed in individuals with higher ADHD PGS at lower levels of prenatal stress suggests that, when the socioeconomic context is favorable during pregnancy, this genetic vulnerability accelerates cortical thinning during adolescence.

Importantly, this interaction was not specific to ADHD genetic vulnerability, as we also observed this finding using the major depression PGS. The low specificity of these PGSs could explain the similar findings observed (Neumann et al., 2022). In contrast, the stronger interactions observed on caudate volume change when using the two alternative PGSs instead of the ADHD PGS suggest that this brain structure may be more sensitive to the specific genetic vulnerability to major depression and schizophrenia. Sensitivity analyses demonstrated that ADHD medication had no impact on our results. In GUSTO, we observed one interaction, but it was not significant after multiple testing corrections. In this case, it was on the right DLPFC cortical thickness change, and it was in the same direction as our finding on mean cortical thickness change in Generation R. The interaction was also driven by prenatal adversity. Therefore, there was some consistency in the findings using two heterogeneous cohorts in terms of genetic ancestry, instruments used, and outcome measurement age periods. A previous study using data from GUSTO reported interactions between prenatal stress and genetic risk for major depression disorder on neonatal structural brain metrics (Qiu et al., 2017). Potential explanations for not finding statistically significant interactions between prenatal stress and ADHD PGS on brain outcomes rate of change in GUSTO could be the smaller sample size, the younger age, the genetic ancestry, or the fact that the A factor score was less comprehensive in GUSTO than in Generation R.

The 3-way interaction models suggested that no evident interactions existed between prenatal stress, child sex, and ADHD PGS on the rate of change in the selected brain outcomes. Some interactions were significant before correcting for multiple testing, but they were not consistent between cohorts. The added value of the 3-way interaction models over 2-way interaction models is that they capture the individual influence of each component on the outcome, whereas dependent on one another (Jolicoeur-Martineau et al., 2019). However, larger samples are needed to be able to draw conclusions.

### 4.1 | Strengths and limitations

The main limitation of this study is our low power to detect small effects, characteristic of gene by environment interaction models. Thus, our results must be interpreted with caution. The probability of overestimated significant estimates and false positives going in the wrong direction is higher in these situations (Gelman & Carlin, 2014). On the other hand, the use of PGS, which summarizes genetic information, as opposed to individual SNPs, allowed us to garner more power and increase the effect size. The disadvantage of using PGS instead of individual SNPs in gene-environment models is that the SNPs included in the PGS may not be relevant for the specific interactions that we were testing. Another limitation of PGS is the selection of the *p* value threshold. The approach we took could lead to overfitting problems, as we selected the best threshold on the same sample used for the main analyses. Furthermore, there is a potential residual confounding by maternal genetics, which may be related to both the genetics of the child and the prenatal stress.

We used latent constructs that covered different areas or sources of stress during pregnancy that we were able to analyze jointly and, at the same time, being able to identify their specific contributions. We did not test the potential impact of postnatal stress in this study. Because prenatal and postnatal stresses are likely related, we are not able to confirm that the observed interactions are exclusively due to prenatal stress.

We also included two MRI assessments, covering two key developmental periods, which is quite unique for this type of studies. Nevertheless, some outcomes, such as surface area, did not show substantial change during the study period, limiting our ability to test the role of subtle interactions on development. The different ancestry background and ages of the two cohorts made the results difficult to compare. However, the inclusion of non-Caucasian populations is rare in this research field and is a strength of this study. Finally, the different characteristics between the included and the excluded participants (i.e., higher maternal education in the included sample) in Generation R may have limited our ability to detect associations. Therefore, the inclusion of more disadvantaged populations is needed.

## 5 | CONCLUSIONS

In conclusion, we found (1) no interactions between prenatal stress and sex on brain development in regions previously associated with ADHD and (2) an interaction between prenatal stress and ADHD PGS on mean cortical thickness annual rate of change during adolescence (i.e., in individuals with higher ADHD PGS, higher prenatal stress was associated with less cortical thinning, whereas in individuals with lower ADHD PGS, higher prenatal stress was associated with a greater cortical thinning). We did not find any interaction in the explorative 3-way interaction models that included prenatal stress, sex, and ADHD PGS. These results could be used as reference for defining hypotheses in future studies.

## Supplementary Material

Supplement / Supporting Information

## Figures and Tables

**TABLE 1:** Characteristics of the participants.

	GUSTO study			Generation R study		
	Included sample (*n* = 113)	Excluded sample^a^ (*n* = 175)	Original sample^b^ (*n* = 1,213)	Included sample (*n* = 433)	Excluded sample^a^ (*n* = 266)	Original sample^b^ (n = 9,202)
Sex (% girls)	56.64	46.86	35.61	49.88	50.00	48.46
Missings (%)	.00	.00	23.5	.00	.00	1.68
Maternal education						
None, primary or secondary(%)	39.82	32.00	27.86	32.56	31.95	51.31*
Higher (%)	58.41	66.86	57.46	66.51	67.29	35.27
Missings (%)	1.77	1.14	14.67	.92	.75	13.41
Maternal mood problems during pregnancy^c^ (mean ± SD)	8.51 ± 6.34	9.24 ± 7.22	8.51 ± 6.17	.16 ± .2	.18 ± .21	.32 ± .4*
Missings (%)	2.65	3.43	35.04	8.08	4.51	34.03
CBCL attention problems T score^d^	53.11 ± 4.64	54.39 ± 5.49	54.24 ± 5.77	53.44 ± 4.74	54.28 ± 5.38*	54.12 ± 5.63*
Missings (%)	28.32	33.14	70.98	6.47	7.52	54.00

*Note:* Chi-square test was used to compare the frequencies of the categorical variables between groups of participants (included sample vs no MRI data, and included sample vs excluded sample) in each cohort, and Wilcoxon rank sum test was used to compare the continuous values between groups of participants in each cohort.

Abbreviations: CBCL, Child Behavior Checklist; GUSTO, Growing Up in Singapore Towards healthy Outcomes.

a Low-quality MRI data in at least one visit.

b Missing genetic, prenatal stress, MRI data in at least one visit (in GUSTO and Generation R) and/or non-European ancestry (in Generation R).

c Maternal mood problems were assessed using the Beck Depression Inventory-II (total score prorated) in GUSTO and the Brief Symptom Inventory (Global Severity Index) in Generation R.

d The CBCL was administered at 4 years old in GUSTO and at 10 years old in Generation R.

* *p* value < .05.

**TABLE 2:** Sex by environment (E, A and M factors) interaction models estimates.

				SpecificEcontributions		
Outcomes^a^	Sex	*E*	Sex:*E*	M factor	A factor	*R* ^2^
*GUSTO*						
**Caudate volume**^b^ **change**	0.415*	−0.330	0.210	0.814	0.186	−13.896
**Putamen volume**^b^ **change**	0.203	−0.057	−0.086	0.840	−0.160	−0.260
**Cerebellar volume**^b^ **change**	−0.093	0.236	−0.375	−0.524	0.476	−0.513
**Cortical thickness change**	0.159	0.289	−0.539	−0.533	0.467	−7.295
**Right DLPFC CT change**	0.193	0.098	−0.285	−0.152	0.848	−0.698
**Right VLPFC CT change**	0.183	0.356	−0.465	−0.425	0.575	−0.233
**Surface area change**	0.439*	−0.122	−0.093	0.166	0.834	−6.553
**Right DLPFC SA**^c^ **change**	−0.018	0.025	0.124	0.264	0.736	−12.295
**Right VLPFC SA**^c^ **change**	−0.280	0.267	−0.095	0.565	0.435	−2.056
**Total brain volume change**	0.442*	0.081	−0.356	−0.347	0.653	−13.792
*Generation R*						
**Caudate volume**^b^ **change**	−0.038	**−0.280** **	0.149	−0.253	0.747**	−0.011
**Putamen volume**^b^ **change**	−0.133	**−0.215** **	0.125	0.080	0.920**	−0.006
**Cerebellar volume**^b^ **change**	−0.229*	−0.172	−0.090	−0.456*	0.544*	−0.041
**Cortical thickness change**	**−0.314** **	−0.077	0.136	0.550	0.450	−0.029
**Right DLPFC CT change**	−0.163	−0.245	0.186	−0.512	0.488	−0.031
**Right VLPFC CT change**	−0.077	0.102	0.028	0.721	−0.279	−0.041
**Surface area change**	**−0.336** ***	0.070	0.006	0.506	−0.494	0.003
**Right DLPFC SA**^c^ **change**	0.008	−0.052	0.180	0.673	0.327	−0.030
**Right VLPFC SA**^c^ **change**	0.181	−0.252*	0.130	−0.427	0.573*	−0.024
**Total brain volume change**	**−0.434** ***	0.057	0.035	0.796	−0.204	0.029

*Note:* Reference = boys. Models adjusted for the first three (GUSTO) or four (Generation R) principal components from GWAS principal component analysis to account for genetic ancestry.

Abbreviations: CT, cortical thickness; DLPFC, dorsolateral prefrontal cortex; GUSTO, Growing Up in Singapore Towards healthy Outcomes; SA, surface area; VLPFC, Ventrolateral prefrontal cortex.

a Annual rates of change residualized on age at baseline scan and normalized using rank-based inverse normal transformation.

b Adjusted for intracranial volume (ratios).

c Adjusted for total surface area (ratios).

“***” 0.001

“**” 0.01

“*” 0.05. ***p* < .006** (Threshold for effective number of tests).

**TABLE 3:** Gene (G, attention-deficit/hyperactivity disorder [ADHD] polygenic risk [PGS]) by environment (E, A and M factors) interaction models estimates.

Outcomes^a^	ADHDPGS threshold	*G*	*E*	*G:E*	Specific E contributions		R^2^
					M factor	A factor	
*GUSTO*							
**Caudate volume**^b^ **change**	0.05	−0.142	−0.119	0.165	0.130	0.870	−7.871
**Putamen volume**^b^ **change**	0.001	−0.154	−0.153	0.202	0.667	−0.333	−0.127
**Cerebellar volume**^b^ **change**	0.2	−0.019	0.018	0.288	0.589	−0.411	−0.099
**Cortical thickness change**	0.1	0.103	−0.099	0.233	−0.392	0.608	−3.505
**Right DLPFC CT change**	0.001	0.156	−0.125	0.255*	0.097	0.903*	−0.115
**Right VLPFC CT change**	1.00E-05	−0.041	0.129	0.259	−0.405	0.595	−0.087
**Surface area change**	1.00E-08	0.099	−0.161	−0.071	0.012	0.988	−5.580
**Right DLPFC SA**^c^ **change**	0.001	0.069	0.046	0.200	0.018	0.982*	−11.112
**Right VLPFC SA**^c^ **change**	0.0001	0.098	0.183	0.095	0.837	0.163	−0.481
**Total brain volume change**	0.05	0.092	0.135	−0.258	0.500	−0.500	−10.548
*Generation R*							
**Caudate volume**^b^ **change**	0.2	−0.039	**−0.154** **	0.098*	0.161	0.839**	−0.006
**Putamen volume**^b^ **change**	1.00E-06	0.069	**−0.172** **	−0.040	0.276	0.724*	0.005
**Cerebellar volume**^b^ **change**	0.2	−0.124**	0.193*	0.051	0.457*	−0.543*	−0.027
**Cortical thickness change**	1.00E-07	−0.043	−0.061	**0.216** ***	−0.151	0.849***	−0.002
**Right DLPFC CT change**	1.00E-07	−0.048	−0.194*	0.167*	−0.517*	0.483*	−0.008
**Right VLPFC CT change**	1.00E-05	0.082	−0.022	0.130*	0.176	0.824*	−0.032
**Surface area change**	1.00E-06	0.078	0.095	−0.059	0.610	−0.390	0.012
**Right DLPFC SA**^c^ **change**	1.00E-06	0.069	0.080	0.017	0.585	−0.415	−0.022
**Right VLPFC SA**^c^ **change**	0.05	−0.047	−0.097*	0.122**	0.002	0.998**	0.000
Total brain volume change	0.0001	0.093	−0.035	0.107	−0.138	0.862	0.025

*Note:* Models adjusted for sex and the first three (GUSTO) or four (Generation R) principal components from GWAS principal component analysis to account for genetic ancestry.

Abbreviations: CT, cortical thickness; DLPFC, dorsolateral prefrontal cortex; GUSTO, Growing Up in Singapore Towards healthy Outcomes; SA, surface area; VLPFC, ventrolateral prefrontal cortex.

a Annual rates of change residualized on age at baseline scan and normalized using rank-based inverse normal transformation.

b Adjusted for intracranial volume (ratios).

c Adjusted for total surface area (ratios).

“***” 0.001

“**” 0.01

“*” 0.05. ***p* < .006** (threshold for effective number of tests).

## Data Availability

The data that support the findings of this study are available on request from the corresponding author. The data are not publicly available due to privacy or ethical restrictions.
