# Supplementary material for: Examining the interaction between prenatal stress and polygenic risk for attention-deficit/hyperactivity disorder on brain growth in childhood: Findings from the DREAM BIG consortium
Source: Dev Psychobiol. Author manuscript; Available in PMC 2025 Mar 14. (PMC11907413; doi:10.1002/dev.22481)
Supplement: Supplement / Supporting Information [file NIHMS2052570-supplement-Supplement___Supporting_Information.docx]

**Supplementary Material**

[Figure S1. Flowchart of the GUSTO Study sample 3](#_Toc154668527)

[Figure S2. Flowchart of the Generation R Study sample 4](#_Toc154668528)

[Appendix S1. Items used for the calculation of the A factor score in GUSTO 5](#_Toc154668529)

[Appendix S2. Items used for the calculation of the A factor score in Generation R 6](#_Toc154668530)

[Appendix S3. Items used for the calculation of the M factor score in GUSTO 8](#_Toc154668531)

[Appendix S4. Items used for the calculation of the M factor score in Generation R 10](#_Toc154668532)

[Appendix S5. Confirmatory factor analysis 11](#_Toc154668533)

[Table S1. Model fit indices from confirmatory factor analyses 12](#_Toc154668534)

[Appendix S6. Genetic data quality assessment and imputation 13](#_Toc154668535)

[Appendix S7. MRI data acquisition and quality assessment 14](#_Toc154668536)

[Figure S3. Distribution of PGS in GUSTO 16](#_Toc154668537)

[Figure S4. Distribution of PGS in Generation R 17](#_Toc154668538)

[Figure S5. Correlations among the polygenic scores calculated at different p value thresholds in GUSTO 18](#_Toc154668539)

[Figure S6. Correlations among the polygenic scores calculated at different p value thresholds in Generation R 19](#_Toc154668540)

[Figure S7. Distribution of A and M factor scores in GUSTO 20](#_Toc154668541)

[Figure S8. Distribution of A and M factor scores in Generation R 21](#_Toc154668542)

[Figure S9. Brain outcome measures in relation to the age at scan (years) in GUSTO 22](#_Toc154668543)

[Figure S10. Brain outcome measures in relation to the age at scan (years) in Generation R 23](#_Toc154668544)

[Figure S11. Unadjusted brain outcome measures in relation to the age at scan (years) in GUSTO 24](#_Toc154668545)

[Figure S12. Unadjusted brain outcome measures in relation to the age at scan (years) in Generation R 25](#_Toc154668546)

[Figure S13. Distribution of the brain annual change values residualized for the age at baseline scan before normalization in GUSTO 26](#_Toc154668547)

[Figure S14. Distribution of the brain annual change values residualized for the age at baseline scan before normalization in Generation R 27](#_Toc154668548)

[Figure S15. Correlations among residualized and normalized brain change values in GUSTO 28](#_Toc154668549)

[Figure S16. Correlations among residualized and normalized brain change values in Generation R 29](#_Toc154668550)

[Table S2. Sex by environment (E) interaction models estimates (separate models for A and M factors) 30](#_Toc154668551)

[Table S3. Gene (G, ADHD PGS) by environment (E) interaction models estimates (separate models for A and M factors) 31](#_Toc154668552)

[Table S4. Gene (G, Major Depression and Schizophrenia PGS) by environment (E, A and M factors) interaction models estimates 32](#_Toc154668553)

[Table S5. Gene (G, ADHD PGS) by environment (E, A and M factors) interaction models estimates excluding children taking ADHD medication (n=17) in Generation R 33](#_Toc154668554)

[Table S6. Gene (G, ADHD PGS) by environment (E, A and M factors) by sex interaction models estimates 34](#_Toc154668555)

Genetic data (n=933)

Prenatal environment (n=842)

Usable FreeSurfer reconstruction

(n=195)

Usable FreeSurfer reconstruction

(n=218)

Structural MRI at 4 years old (n=287)

Structural MRI at 6 years old (n=334)

Incidental finding (n=2)

Unusable FreeSurfer reconstruction (n=90)

Incidental finding (n=4)

Unusable FreeSurfer reconstruction (n=112)

Overlap=113

GUSTO Study

# Figure S1. Flowchart of the GUSTO Study sample

Genetic data, European-ancestry participants (n=2,418)

Prenatal environment (n=2,344)

Usable FreeSurfer reconstruction

(n=1,074)

Usable FreeSurfer reconstruction

(n=738)

Structural MRI at 10 years old (n=1,253)

Structural MRI at 14 years old (n=853)

Different T1 acquisition (n=8)

Incidental finding (n=5)

Unusable FreeSurfer reconstruction (n=166)

Incidental finding (n=2)

Unusable FreeSurfer reconstruction (n=113)

Overlap=433

Generation R Study

# Figure S2. Flowchart of the Generation R Study sample

# Appendix S1. Items used for the calculation of the A factor score in GUSTO

| **Latent subfactors** | **Dichotomous items** | **Timing** | **Instrument name** |
| --- | --- | --- | --- |
| **Life stress** | Death of a family member within 10 months before the birth of child | 24 months | Parent’s experiences in loss Questionnaire |
|  | Change of job due to health problems during pregnancy | 26-28 week | General pregnancy questions |
|  | Unplanned pregnancy |  |  |
| **Contextual stress** | Mother’s monthly income (< 999 SGD) | 11-12 week |  |
|  | Household monthly income (< 999 SGD) |  |  |
|  | House inadequacy (1-2 room flat) |  |  |
| **Personal stress** | Young age (<19) |  |  |
|  | Smoking during pregnancy | 26-28 week |  |
|  | Education (secondary or lower) | 11-12 week |  |
| **Interpersonal stress** | Among family and friends, is there someone who would help you in times of need? | 26-28 week | LYDON Maternal Health and Well Being Questionnaire |
|  | Among family and friends, is there someone you can confide in or talk freely about your problems? |  |  |
|  | During the last two weeks, did it happen that you needed something like help to babysit children, run errands, or clean the house? If yes, did anyone help you? |  |  |
|  | During the last two weeks, did it happen that you needed information, input or guidance in a particular situation for you or another member of your family? If yes, did anyone help you? |  |  |
|  | During the last two weeks, did it happen that you needed to talk to someone about something personal or intimate? If yes, did you find anyone to help you? |  |  |
|  | During the last two weeks, did you feel that you needed someone to give you feedback, that is, to approve you or tell you that you have made the right choice or right decision? If yes, did you find anyone to help you? |  |  |
|  | During the last 3 months, did you receive help for your household tasks/chores? |  |  |
|  | Do you currently have a live-in domestic helper? |  | Questions on domestic helper |
|  | Single mother (or divorced, or married not living with husband) | 11-12 week | General pregnancy questions |

# Appendix S2. Items used for the calculation of the A factor score in Generation R

| **Latent subfactors** | **Dichotomous items** | **Timing (weeks gestation)** | **Instrument name** |
| --- | --- | --- | --- |
| **Life stress** | Death of a child | 20-25 weeks | Social Readjustment Rating Scale (SRRS) |
|  | Death of a partner |  |  |
|  | Death of a friend or relative |  |  |
|  | Serious illness of child |  |  |
|  | Serious illness of a close relative |  |  |
|  | Job loss |  |  |
|  | Change of residence |  |  |
|  | Personal illness (moderate or poor health) | 20-25 weeks | 12-Item Short Form Health Survey (SF-12) |
|  | Problems at work or in studies | 20-25 weeks | Long Lasting Difficulties Questionnaire (LLDQ) |
|  | Unplanned pregnancy | 12-20 weeks | General pregnancy questions - Generation R |
|  | Chromosomal abnormalities testing | 30 weeks |  |
|  | Dissatisfaction with obstetric care |  |  |
|  | Admission to a hospital for >24 hours |  |  |
|  | Victim of robbery, theft, physical abuse or rape within the last 12 months | 20-25 weeks | General life events questions - Generation R |
| **Contextual Stress** | Lack of major appliances (heating, washer, refrigerator) within the last 12 months | 30 weeks | General sociodemographic questions - Generation R |
|  | Housing defects (e.g. draughts, dampness) within the last 12 months |  |  |
|  | Financial difficulties (e.g. difficulties paying rent) within the last 12 months |  |  |
|  | House inadequacy (e.g., size, lack of privacy) | 20-25 weeks | LLDQ |
|  | Major financial problem (e.g. insufficient income) |  |  |
|  | Downturn in financial situation | 20-25 weeks | SRRS |
| **Personal Stress** | Having a criminal record | 20-25 weeks | General questions about public order offenses - Generation R |
|  | Violent interpersonal offenses |  |  |
|  | Public order offenses |  |  |
|  | Addicted to alcohol or drugs, excluding nicotine, within the past year | 20-25 weeks | General substance use questions - Generation R |
|  | Has not finished upper secondary school | enrollment | General sociodemographic questions - Generation R |
|  | Young age (<19 years) |  |  |
| **Interpersonal Stress** | Difficulties with partner | 20-25 weeks | LLDQ |
|  | Difficulties with family or friends |  |  |
|  | Difficulties in contact with others |  |  |
|  | Single mother | 12-20 weeks | General sociodemographic questions - Generation R |
|  | Family size (>3 people) | 20-25 weeks |  |
|  | Family affection problems | 20-25 weeks | Family Assessment Device (FAD) |
|  | Difficulty in making plans |  |  |
|  | Disapproval of others |  |  |
|  | Difficulty in talking about sadness |  |  |
|  | Avoidance of talking about problems |  |  |
|  | Feelings of not being accepted |  |  |
|  | Unpleasant and painful feelings |  |  |
|  | Inability to solve problems |  |  |
|  | Decision-making problems |  |  |
|  | Distrust between family members |  |  |
|  | Conflicts between family members |  |  |
|  | Family support problems |  |  |
|  | Divorce in the past year | 20-25 weeks | SRRS |

# Appendix S3. Items used for the calculation of the M factor score in GUSTO

| **Latent subfactors** | **Continuous items** | **Timing (weeks gestation)** | **Instrument name** |
| --- | --- | --- | --- |
| **Anxiety/ depression** | I feel calm | 26-28 week | State Trait Anxiety Inventory (STAI) (state anxiety items) |
|  | I feel secure |  |  |
|  | I am tense |  |  |
|  | I feel strained |  |  |
|  | I feel at ease |  |  |
|  | I feel upset |  |  |
|  | I am presently worrying over possible misfortunes |  |  |
|  | I feel satisfied |  |  |
|  | I feel frightened |  |  |
|  | I feel comfortable |  |  |
|  | I feel self-confident |  |  |
|  | I feel nervous |  |  |
|  | I am jittery |  |  |
|  | I feel indecisive |  |  |
|  | I am relaxed |  |  |
|  | I feel content |  |  |
|  | I am worried |  |  |
|  | I feel confused |  |  |
|  | I feel steady |  |  |
|  | I feel pleasant |  |  |
|  | Sadness |  | Beck Depression Inventory-II (BDI2) |
|  | Pessimism |  |  |
|  | Past Failure |  |  |
|  | Loss of Pleasure |  |  |
|  | Guilty Feelings |  |  |
|  | Punishment Feelings |  |  |
|  | Self-Dislike |  |  |
|  | Self-Criticalness |  |  |
|  | Suicidal Thoughts or Wishes |  |  |
|  | Crying |  |  |
|  | Loss of Interest |  |  |
|  | Indecisiveness |  |  |
|  | Worthlessness |  |  |
|  | Irritability |  |  |
|  | In the past 7 days, I have been able to laugh and see the funny side of things |  | Edinburgh Postnatal Depression Scale (EPDS) |
|  | In the past 7 days, I have looked forward with enjoyment to things |  |  |
|  | In the past 7 days, I have blamed myself unnecessarily when things went wrong |  |  |
|  | In the past 7 days, I have been anxious or worried for no good reason |  |  |
|  | In the past 7 days, I have felt scared or panicky for no very good reason |  |  |
|  | In the past 7 days, things have been getting on top of me |  |  |
|  | In the past 7 days, I have been so unhappy that I have had difficulty sleeping |  |  |
|  | In the past 7 days, I have felt sad or miserable |  |  |
|  | In the past 7 days, I have been so unhappy that I have been crying |  |  |
|  | In the past 7 days, the thought of harming myself has occurred to me |  |  |
| **Somatic** | Agitation |  | BDI2 |
|  | Loss of Energy |  |  |
|  | Changes in Sleeping Pattern |  |  |
|  | Changes in Appetite |  |  |
|  | Concentration Difficulty |  |  |
|  | Tiredness or Fatigue |  |  |
| **Pregnancy worries** | To what extend do you feel committed to having a baby right now? |  | Items from the LYDON Maternal Health and Well Being Questionnaire |
|  | To what extend do you feel enthusiastic about pregnant right now? |  |  |
|  | To what extend does having a baby right now interfere with or compete with other goals in your life? |  |  |
| **Positive wording** | I feel calm |  | STAI |
|  | I feel secure |  |  |
|  | I feel at ease |  |  |
|  | I feel satisfied |  |  |
|  | I feel comfortable |  |  |
|  | I feel self-confident |  |  |
|  | I am relaxed |  |  |
|  | I feel content |  |  |
|  | I feel steady |  |  |
|  | I feel pleasant |  |  |
|  | In the past 7 days, I have been able to laugh and see the funny side of things |  | EPDS |
|  | In the past 7 days, I have looked forward with enjoyment to things |  |  |
|  | To what extend do you feel committed to having a baby right now? |  | LYDON |
|  | To what extend do you feel enthusiastic about pregnant right now? |  |  |

# Appendix S4. Items used for the calculation of the M factor score in Generation R

| **Latent subfactors** | **Continuous items** | **Timing (weeks gestation)** | **Instrument name** |
| --- | --- | --- | --- |
| **Anxiety/ depression** | Feeling suicidal | 20-25 weeks | Brief Symptom Inventory |
|  | Feeling lonely |  |  |
|  | Feeling down |  |  |
|  | Having no interest in anything anymore |  |  |
|  | Feeling of desperation about the future |  |  |
|  | Feeling worthless |  |  |
|  | Nervousness or shaking inside |  |  |
|  | Suddenly getting a fright or feeling anxious |  |  |
|  | Feeling afraid |  |  |
|  | Feeling tense |  |  |
|  | Anxiety or panic attacks |  |  |
|  | Feeling so restless that you cannot sit still |  |  |
| **Somatic** | Dizziness | 20-25 weeks | Brief Symptom Inventory |
|  | Pain in the chest or around the heart |  |  |
|  | Nausea or an upset stomach |  |  |
|  | Difficulty in catching your breath |  |  |
|  | Feeling very hot and then very cold |  |  |
|  | Numbness or tingling feeling somewhere in the body |  |  |
|  | Feeling physically weak |  |  |
| **Pregnancy worries** | I think the pregnancy will take place without special problems | 12-20 weeks | Pregnancy Outcome Questionnaire |
|  | I think my nervousness will have an effect on the baby |  |  |
|  | I am worried about the health of the baby |  |  |
|  | I wonder about what it will be like with the baby after birth |  |  |
|  | I am sorry that I told other people about the pregnancy |  |  |
|  | I feel fully able to cope with the fears related to pregnancy |  |  |
|  | I worry about whether the pregnancy will go well or not |  |  |
|  | I feel overwhelmed by fear about this pregnancy |  |  |
|  | I wonder whether I can look after the baby properly |  |  |
|  | I feel sure I have some influence over the outcome of pregnancy |  |  |
|  | I am entirely preoccupied with thinking about birth and labor |  |  |
|  | I am sure that the baby will be healthy |  |  |
|  | I feel reluctant about making preparations for the baby |  |  |
| **Positive wording** | I think the pregnancy will take place without special problems | 12-20 weeks | Pregnancy Outcome Questionnaire |
|  | I feel fully able to cope with the fears related to pregnancy |  |  |
|  | I feel sure I have some influence over the outcome of pregnancy |  |  |
|  | I am sure that the baby will be healthy |  |  |

# Appendix S5. Confirmatory factor analysis

The construction of the A-factor is based on the cumulative environmental risk score, which has been described in detail elsewhere ^1,2^. Briefly, to compute a cumulative environmental risk score for each participant, the authors first summed risk items to create four conceptually distinct but related risk domains: life events; contextual risks; parental risks; interpersonal risks, which were positively and significantly correlated. Confirmatory factor analysis (CFA) was used to assess internal reliability of the risk domains and to extract one global cumulative risk score for each participant. The prenatal environmental adversity score (A-factor) is a modified version of the above cumulative environmental risk score. Firstly, parental psychopathology items were removed, as these were modeled separately and in more detail inside the M-factor. Secondly, instead of summing the risk items to create the four risk domains before extracting an overall risk factor, risk items were first loaded onto their corresponding risk domains and then the four risk domains were loaded onto an overall A-factor using CFA with a hierarchical second-order structure. CFA were performed using the lavaan R package ^3^. Weighted least square means and variances (WLSMV) were used in all cohorts (due to the presence of dichotomous indicator variables). To maximize the number of observations included in analyses and prevent sampling bias, participants with available data on at least one environmental adversity item were included in analyses. Missing values were estimated using multiple imputation across 40 imputed datasets using the mice package in R ^4^. The extracted A-factor scores were standardized in each of the cohorts. Goodness of fit was evaluated using three indices: the comparative fit index (CFI), Tucker-Lewis index (TLI) and room mean square error of approximation (RMSEA).

The construction of the M-factor was described in detail elsewhere ^5^. Briefly, CFA were sequentially used to identify best fitting and parsimonious model(s). We first examined the simplest single factor model that assumed all prenatal affective symptoms load onto a single dimension; then a series of more complex models with multiple factors which assumed that the various prenatal affective symptoms load onto several more specific but correlated dimensions (defined as per the prevailing literature); and finally a bifactor model that evaluated the existence of a general factor in addition to the specific symptom dimensions, as previously suggested ^6^. We compared a series of increasingly complex models from a simple unifactor model to a bifactor model, in which all symptoms load onto a general factor and to their corresponding specific factor (i.e. depression/anxiety, somatic symptoms, or pregnancy-specific worries). Confirmatory factor analyses were performed using the lavaan R package ^3^. Robust maximum likelihood estimators were used (all continuous indicator variables). To maximize the number of observations included in analyses and prevent sampling bias, participants with available data on at least one psychopathology subscale were included in analyses. Incomplete indicators were handled using full information maximum likelihood. Latent variables were standardized in each of the cohorts. Goodness of fit was evaluated using three indices: the CFI, the TLI and the RMSEA.

# Table S1. Model fit indices from confirmatory factor analyses

|  | **A factor^1^** | | **M factor^2^** | |
| --- | --- | --- | --- | --- |
|  | **GUSTO** | **Generation R** | **GUSTO** | **Generation R** |
| **RMSEA (90% CI)** | 0.054 (0.049, 0.059) | 0.037 (0.037, 0.038) | 0.048 (0.047, 0.050) | 0.037 (0.036, 0.038) |
| **CFI** | 0.803 | 0.863 | 0.846 | 0.931 |
| **TLI** | 0.759 | 0.856 | 0.832 | 0.920 |

^1^ Scaled indices; ^2^robust indices

CFI: comparative fit index; RMSEA: root mean square error of approximation; TLI: Tucker-Lewis index.

# Appendix S6. Genetic data quality assessment and imputation

*GUSTO*

Infant DNA was extracted from cord tissue or blood that has previously described ^7^. The extracted DNA was genotyped using Illumina OmniExpress plus Exome array and the commercially available PsychArray (Illumina). DNA hybridization arrays and scanning were performed by Expression Analysis, Inc. (Morrisville, NC). Data were processed using GenomeStudio Genotyping Module version 1.0 (Illumina, Inc.). Briefly, genotyping calls were made by the GenCall software and genotypes with a GenCall score less than 0.15 are not assigned genotypes. Samples with genotyping call rate <97%, not matching self-reported ethnicity or discrepant in sex or with incongruent offspring-parent relationship (expected PI_HAT=0.5) were removed. For genotype imputation, SNPs with MAF <1%, or fail Hardy-Weinberg Equilibrium at value of p<10−3 were excluded using PLINK version 1.90. The data were aligned to GRCh37 build and further processed before haplotype phasing using SHAPEIT2. We imputed the phased haplotypes using Impute2 and the 1000 Genomes Phase 3 as reference panel. We analyzed 6,254,009 SNPs that passed stringent quality control (minor allele frequency (MAF) >1% and imputation INFO>0.50).

*Generation R*

Infant DNA was extracted from cord tissue or blood that has previously described ^8^. Genotyping was performed using Illumina HumanHap 610 or 660 Quad chips and intensities were obtained from the BeadArray Reader. Genotype calling was performed on normalized intensities using the GenCall software from the GenomeStudio Genotyping Module version 1.1.0.28426 (Illumina, Inc.). A no-call threshold of 0.15 was applied to a manufacturer-provided cluster file. We used a threshold of 97.5% for exclusion of samples. We tested excess heterozygosity, sex accuracy, and relatedness. The QC procedures were applied to the genotyped data using PLINK. QC filters included sample (≥97.5%) and SNP call rates (≥95%), MAF ≥1% and Hardy-Weinberg equilibrium deviations (p<10-7). Data was imputed to the HRC 1.1 reference panel using the Michigan Imputation Server. SNPs with imputation quality (R 2) below 0.80 were excluded, leaving 2,707,736‬ SNPs for analysis.

# Appendix S7. MRI data acquisition and quality assessment

*GUSTO*

In the GUSTO Study, the images were acquired using a 3 Tesla Siemens Skyra scanner (Siemens, Munich, Germany) with a 32-channel head coil. Children went through an MRI home training program prior to the MRI visit and on-site MRI training. Structural T1-weighted images were obtained using a sagittal rapid gradient recalled echo (repetition time = 2000 ms, echo time = 2.08 ms, inversion time = 877 ms, flip angle = 9◦ , matrix = 192 × 192, field of view = 192 mm × 192 mm, slice thickness = 1 mm) ^9^.

At the time of the MRI acquisition, T1 images were evaluated for incidental findings and rated for image quality using a four‐point Likert scale. The quality assessment levels for the scans were: unusable, large motion, minor motion, and no motion. The visual inspection measures used to make this assessment included the sharpness of the gray and white matter interface in the cortex, the presence of ringing in the image, and whole brain coverage. If the initial T1 scan was rated as unusable or poor by the technician running the scanner, the T1 sequence was repeated. Prior to repeating the scan, communication took place between the child and MR technician to make sure that the child was comfortable in the scanner. The usable scans were those rated as having either minor or no motion.

Following Freesurfer guidelines, visual inspection of brain skull stripping, white matter, and pial surfaces was conducted. The manual correction, such as adding control points, based on FreeSurfer guideline was also performed.

*Generation R*

In the Generation R Study, magnetic resonance images were acquired on a study-dedicated 3 Tesla GE Discovery MR750w MRI System (General Electric, Milwaukee, WI, United States) scanner using an 8-channel head coil. No hardware upgrades or major software upgrades have taken place since the study began in 2012 in order to keep the system stable for longitudinal research. After a brief mock scanning session to acclimate the participants to the MRI environment, structural T1-weighted images were obtained using a 3D coronal inversion recovery fast spoiled gradient recalled (IR-FSPGR, BRAVO) sequence using ARC acceleration (repetition time = 8.77 ms, echo time = 3.4 ms, inversion time = 600 ms, flip angle = 10◦ , matrix = 220 × 220, field of view = 220 mm × 220 mm, slice thickness = 1 mm) ^10^.

At the time of the MRI acquisition, T1 images were evaluated for incidental findings and rated for image quality using a six‐point Likert scale ^11^. The quality assessment levels for the scans were: unusable, poor, fair, good, very good, and excellent. The visual inspection measures used to make this assessment included the sharpness of the gray matter and white matter interface on the cortex, the presence of ringing in the image, and whole brain coverage. If the initial T1 scan was rated as unusable or poor by the technician or PhD student running the scanner, the T1 sequence was repeated. Prior to repeating the scan, communication took place between the child and MR technician to make sure that the child was comfortable in the scanner and to remind the child to remain as still as possible.

All FreeSurfer reconstructions, including 2‐D segmentations and 3‐D morphometry were visually inspected using a 3‐point Likert scale with the following levels: “Excellent to Very Good,” “Good to Fair,” and “Poor to Unusable.” For the age-14 visit, some images were assessed by two independent raters and the other, by one rater and an automated QC ratings from an in-house Support Vector Machine algorithm. In case of disagreement, another rater was consulted.


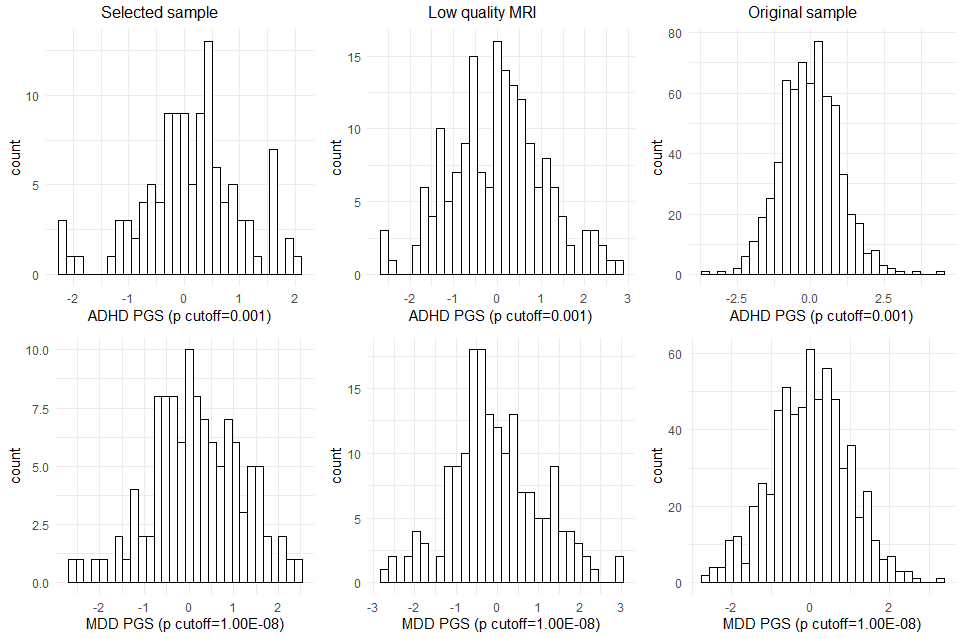


# Figure S3. Distribution of PGS in GUSTO

Selected sample: subjects will all data available (n=133), Low quality MRI: subjects with all data available excluded due to low quality MRI data in at least one visit (n=175), Original sample: subjects with missing prenatal stress and/or MRI data in at least one visit (n=645). ADHD: Attention-deficit/hyperactivity disorder; MDD: Major depression disorder; MRI: magnetic resonance imaging; PGS: Polygenic score


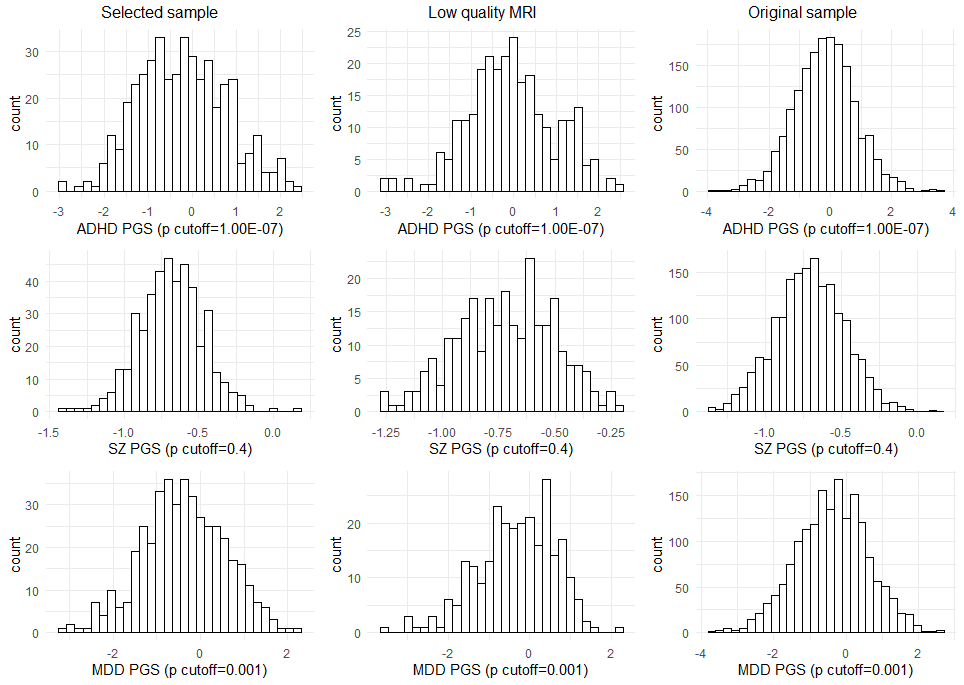


# Figure S4. Distribution of PGS in Generation R

Selected sample: subjects will all data available (n=433), Low quality MRI: subjects with all data available excluded due to low quality MRI data in at least one visit (n=266), Original sample: subjects with missing prenatal stress and/or MRI data in at least one visit (n=1719). ADHD: Attention-deficit/hyperactivity disorder; MDD: Major depression disorder; MRI: magnetic resonance imaging; PGS: Polygenic score; SZ: Schizophrenia


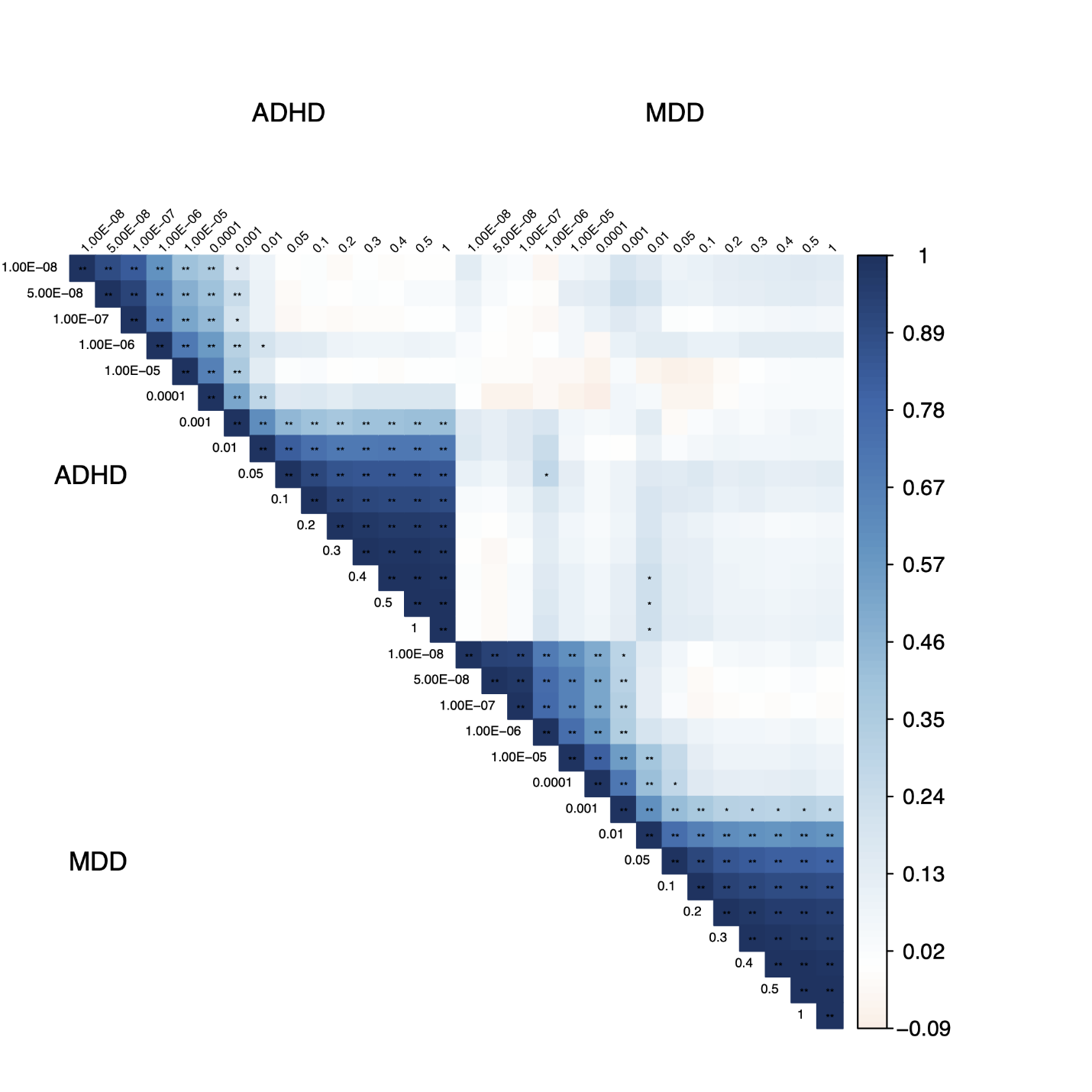


Figure S5. Correlations among the polygenic scores calculated at different p value thresholds in GUSTO

‘**’ 0.001 ‘*’ 0.01. ADHD: Attention-deficit/hyperactivity disorder; MDD: Major depression disorder


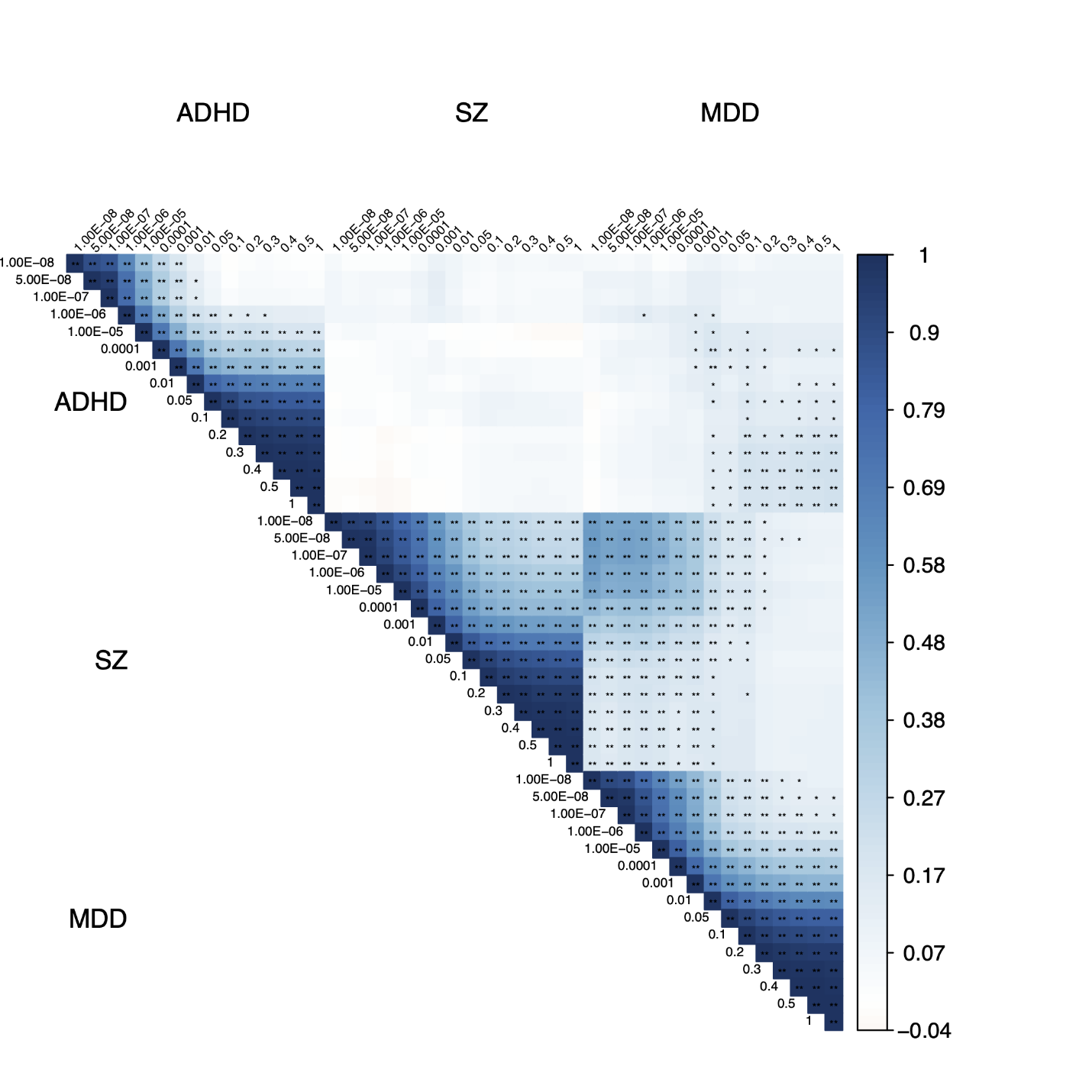


Figure S6. Correlations among the polygenic scores calculated at different p value thresholds in Generation R

‘**’ 0.001 ‘*’ 0.01. ADHD: Attention-deficit/hyperactivity disorder; MDD: Major depression disorder; SZ: Schizophrenia


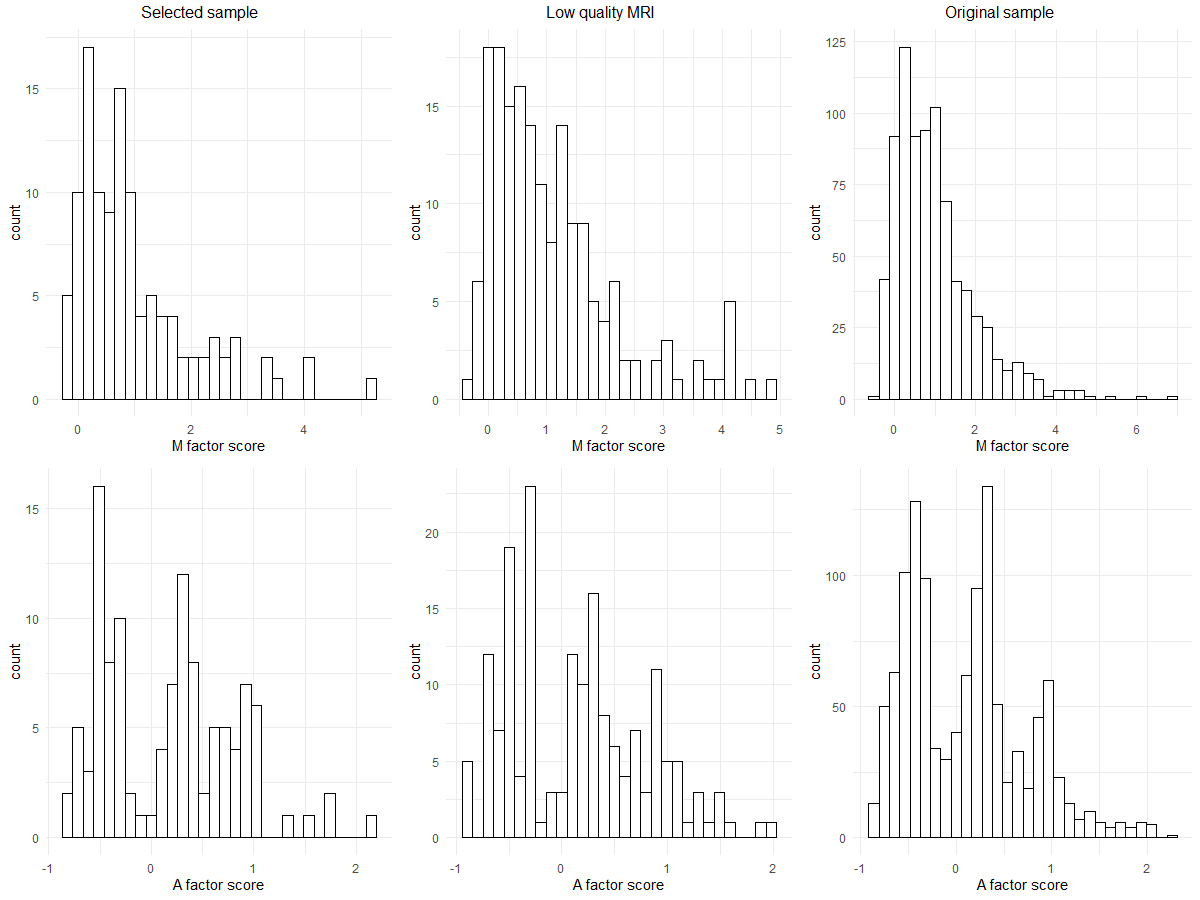


# Figure S7. Distribution of A and M factor scores in GUSTO

Selected sample: subjects will all data available (n=133), Low quality MRI: subjects with all data available excluded due to low quality MRI data in at least one visit (n=175), Original sample: subjects with missing genetic data and/or missing MRI data in at least one visit (n=815 for the M factor, and n=1164 for the A factor). MRI: magnetic resonance imaging


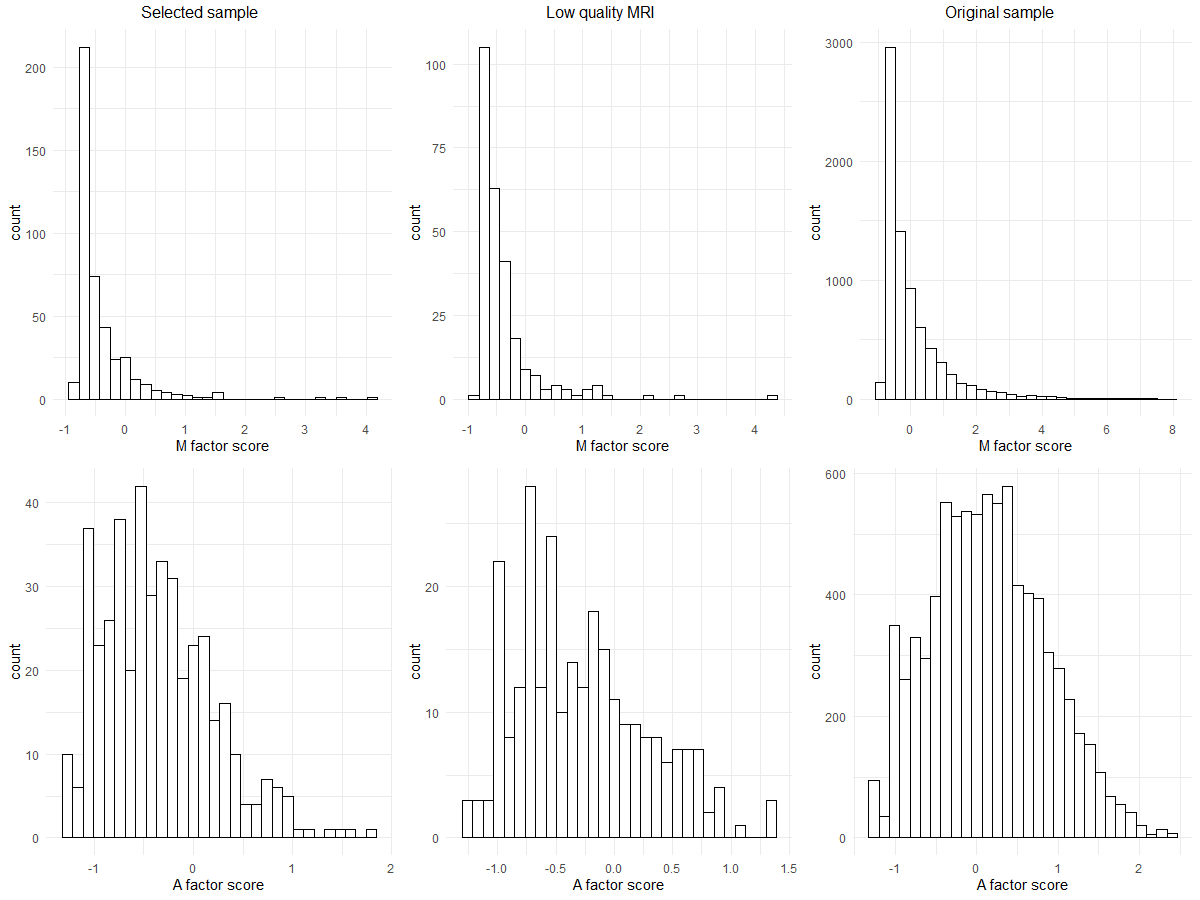


# Figure S8. Distribution of A and M factor scores in Generation R

Selected sample: subjects will all data available (n=433), Low quality MRI: subjects with all data available excluded due to low quality MRI data in at least one visit (n=266), Original sample: subjects with missing genetic data, missing MRI data in at least one visit and/or non-European ancestry (n=7640 for the M factor, and n=8277 for the A factor). MRI: magnetic resonance imaging


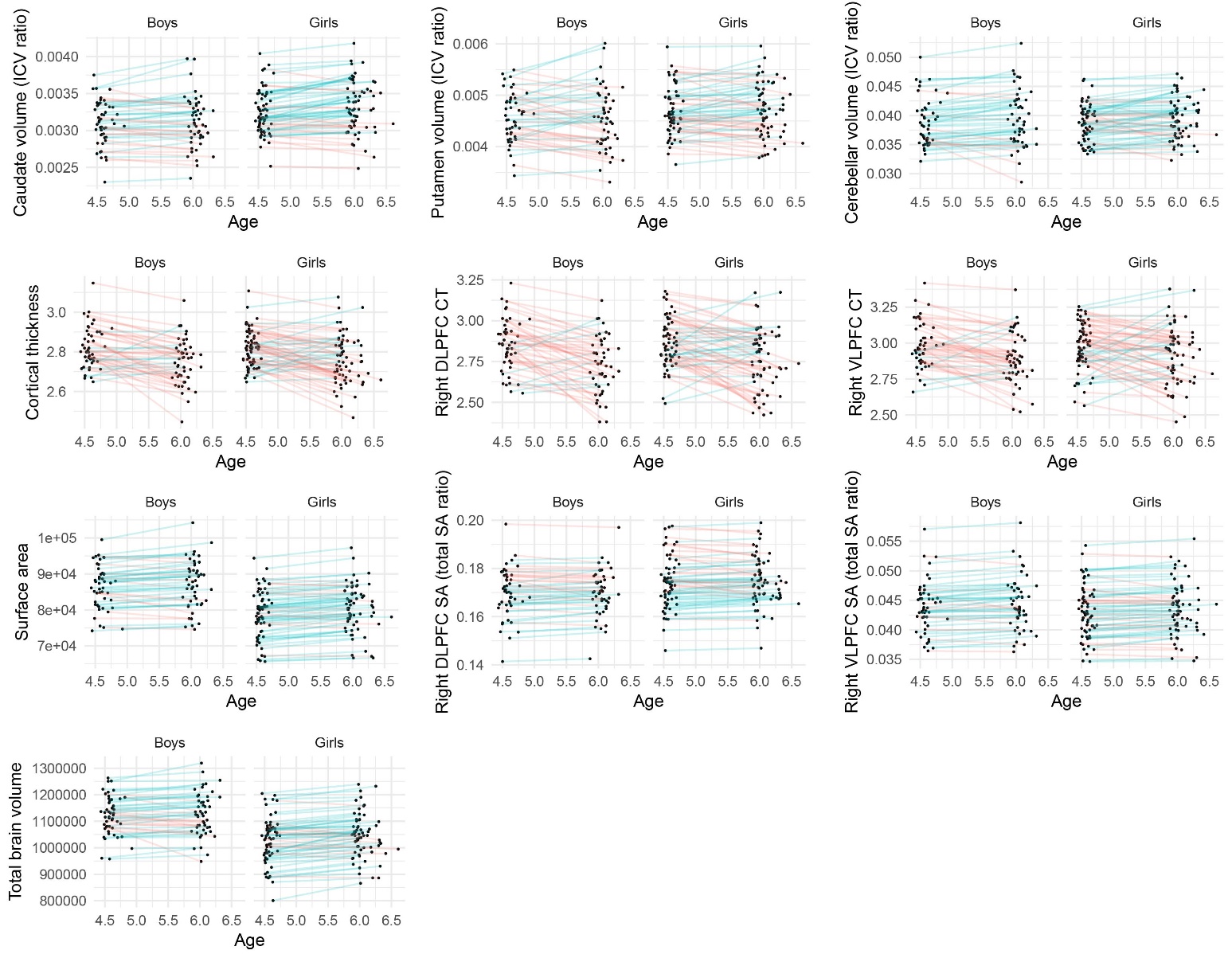


# Figure S9. Brain outcome measures in relation to the age at scan (years) in GUSTO

The observations of each participant are connected using lines (red lines denote a decrease between the two time points, blue lines denote an increase between the two time points). CT: cortical thickness; DLPFC: Dorsolateral prefrontal cortex; ICV: intracranial volume; SA: surface area; VLPFC: Ventrolateral prefrontal cortex.


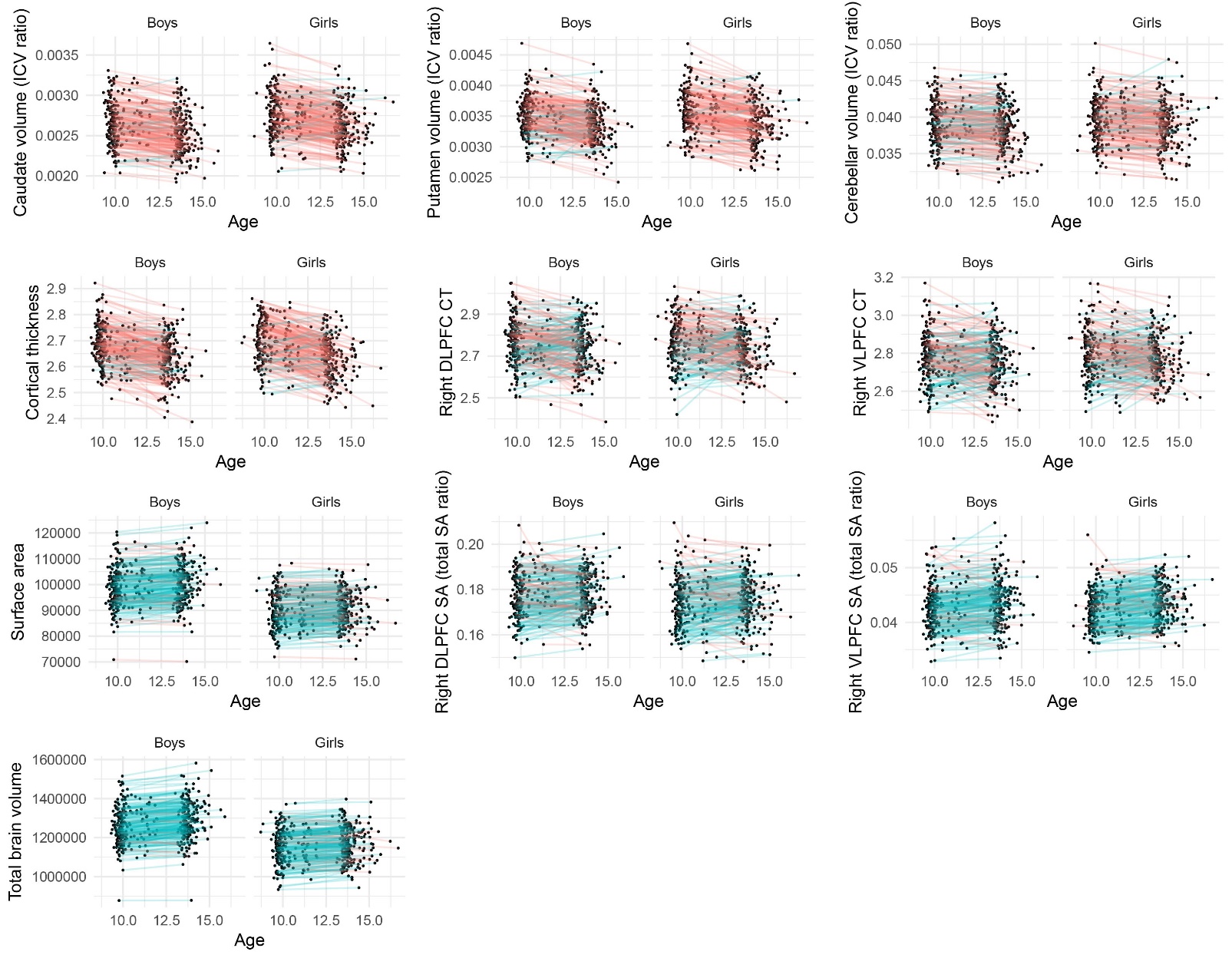


# Figure S10. Brain outcome measures in relation to the age at scan (years) in Generation R

The observations of each participant are connected using lines (red lines denote a decrease between the two time points, blue lines denote an increase between the two time points). CT: cortical thickness; DLPFC: Dorsolateral prefrontal cortex; ICV: intracranial volume; SA: surface area; VLPFC: Ventrolateral prefrontal cortex.


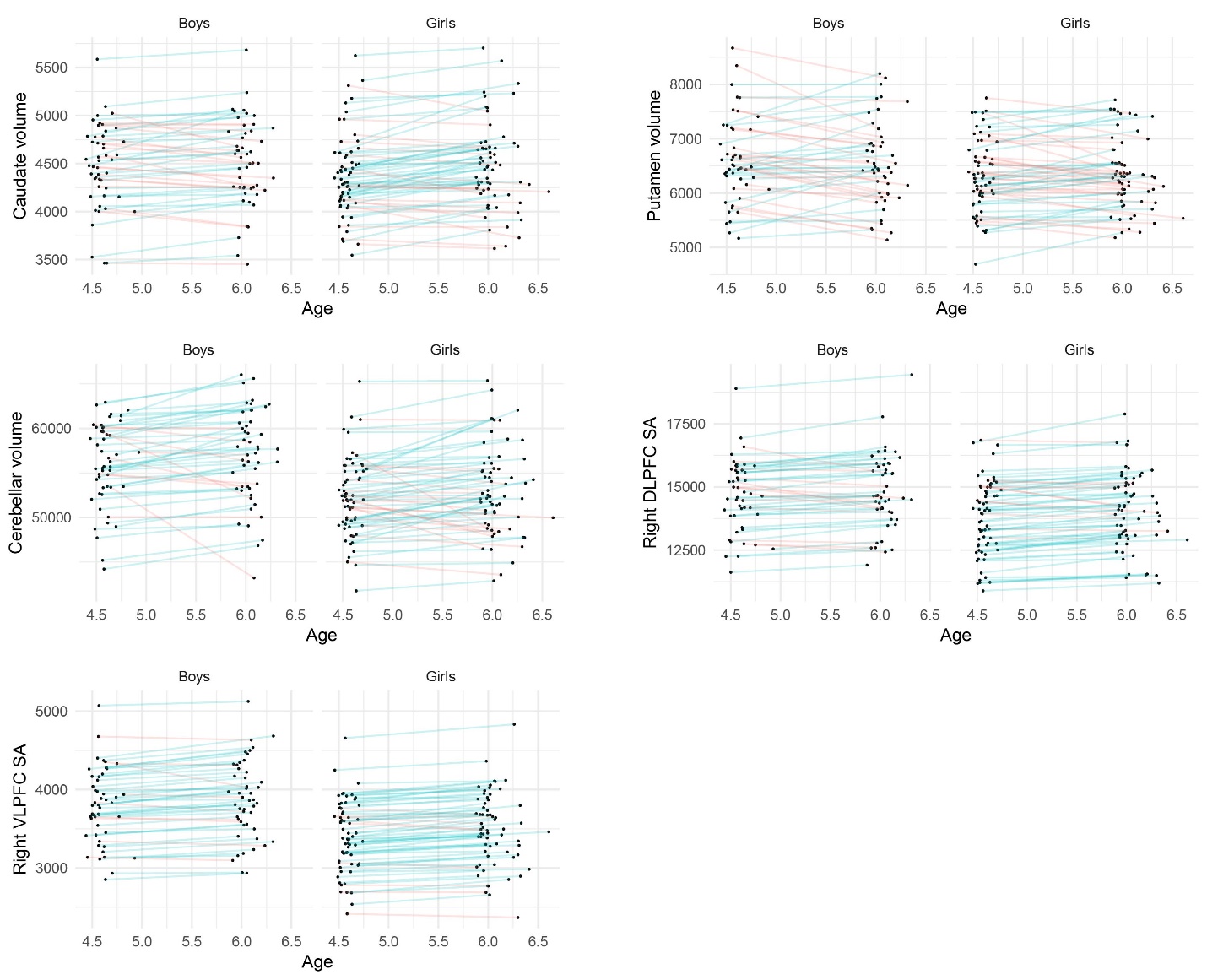


# Figure S11. Unadjusted brain outcome measures in relation to the age at scan (years) in GUSTO

The observations of each participant are connected using lines (red lines denote a decrease between the two time points, blue lines denote an increase between the two time points). DLPFC: Dorsolateral prefrontal cortex; SA: surface area; VLPFC: Ventrolateral prefrontal cortex.


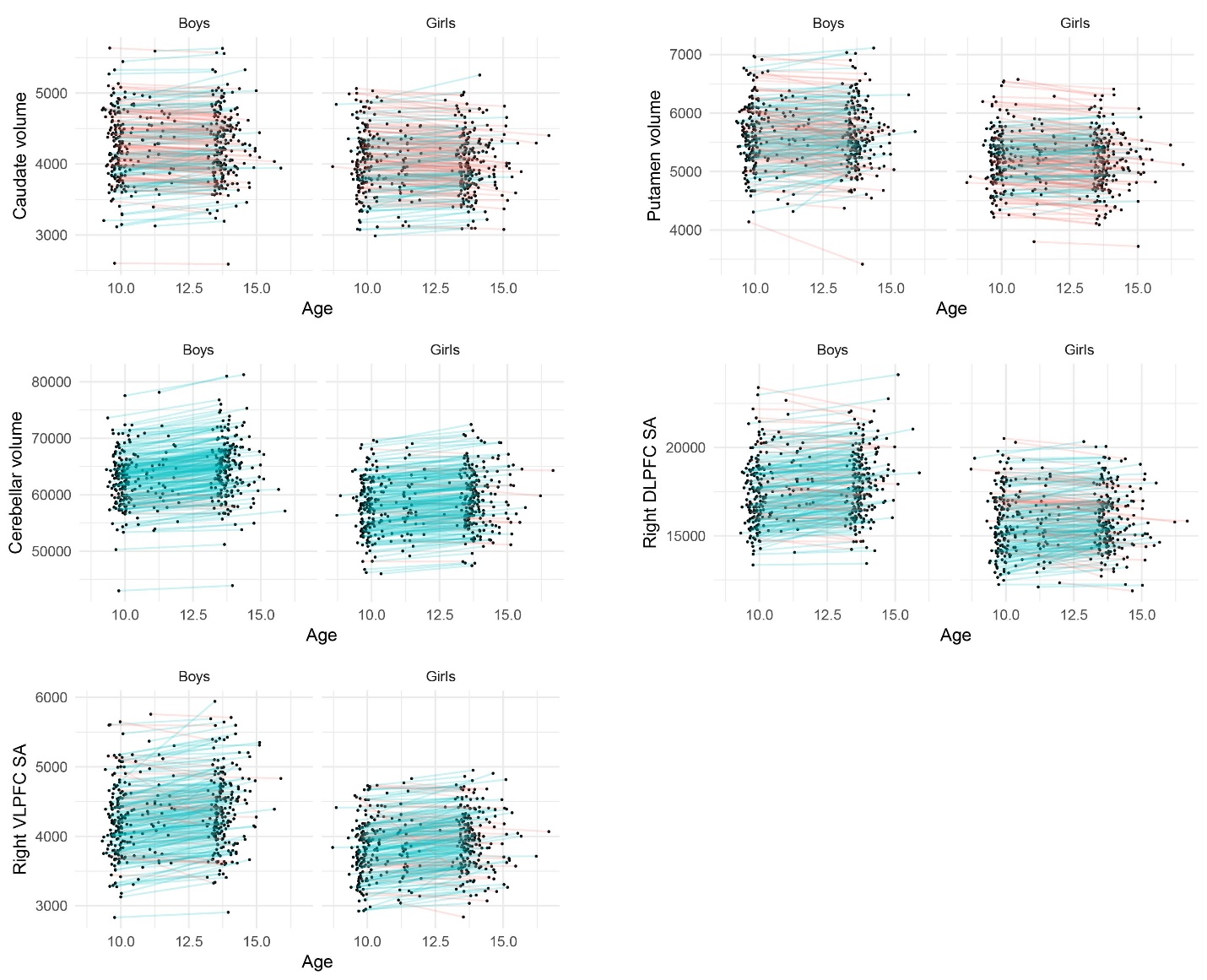


# Figure S12. Unadjusted brain outcome measures in relation to the age at scan (years) in Generation R

The observations of each participant are connected using lines (red lines denote a decrease between the two time points, blue lines denote an increase between the two time points). DLPFC: Dorsolateral prefrontal cortex; SA: surface area; VLPFC: Ventrolateral prefrontal cortex.


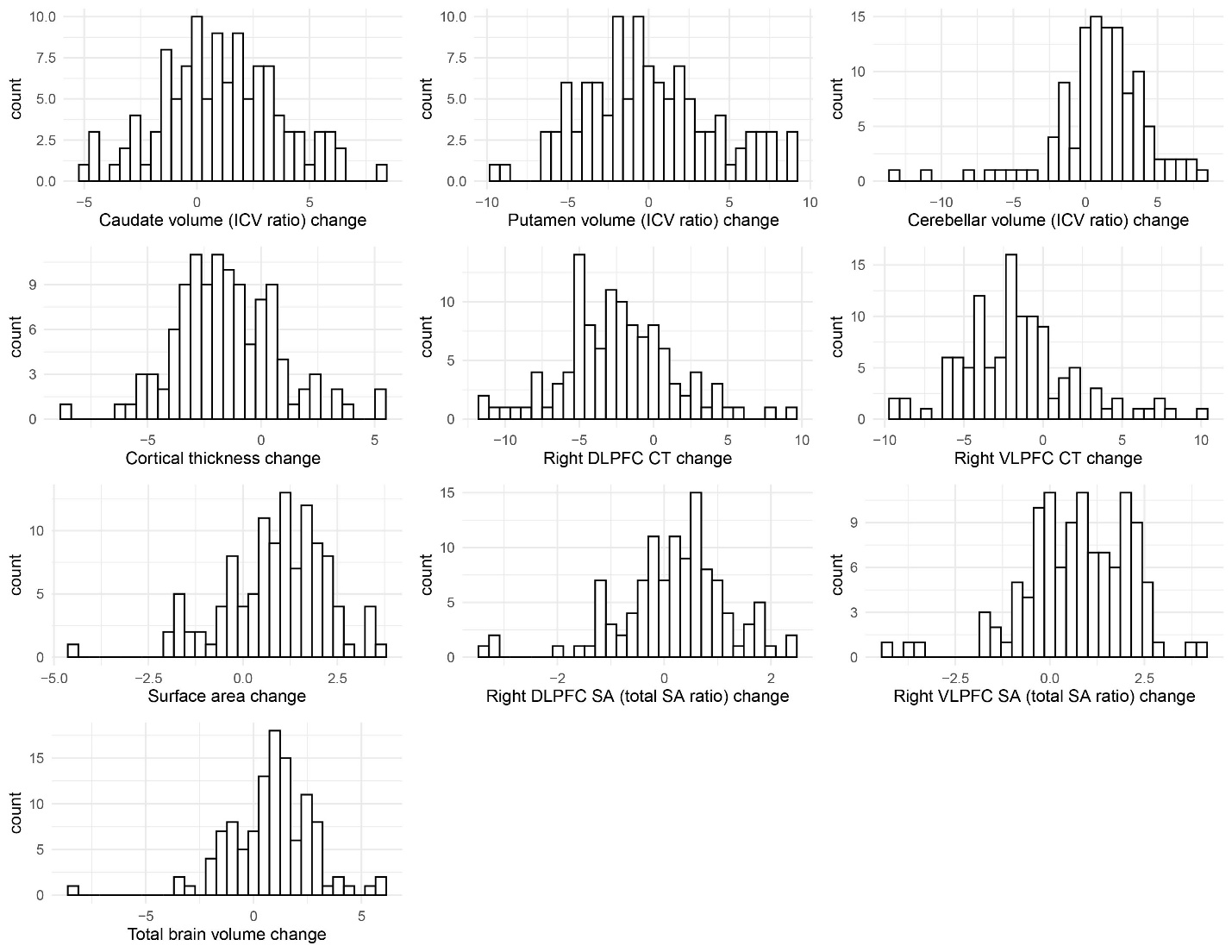


# Figure S13. Distribution of the brain annual change values residualized for the age at baseline scan before normalization in GUSTO

CT: cortical thickness; DLPFC: Dorsolateral prefrontal cortex; ICV: intracranial volume; SA: surface area; VLPFC: Ventrolateral prefrontal cortex.


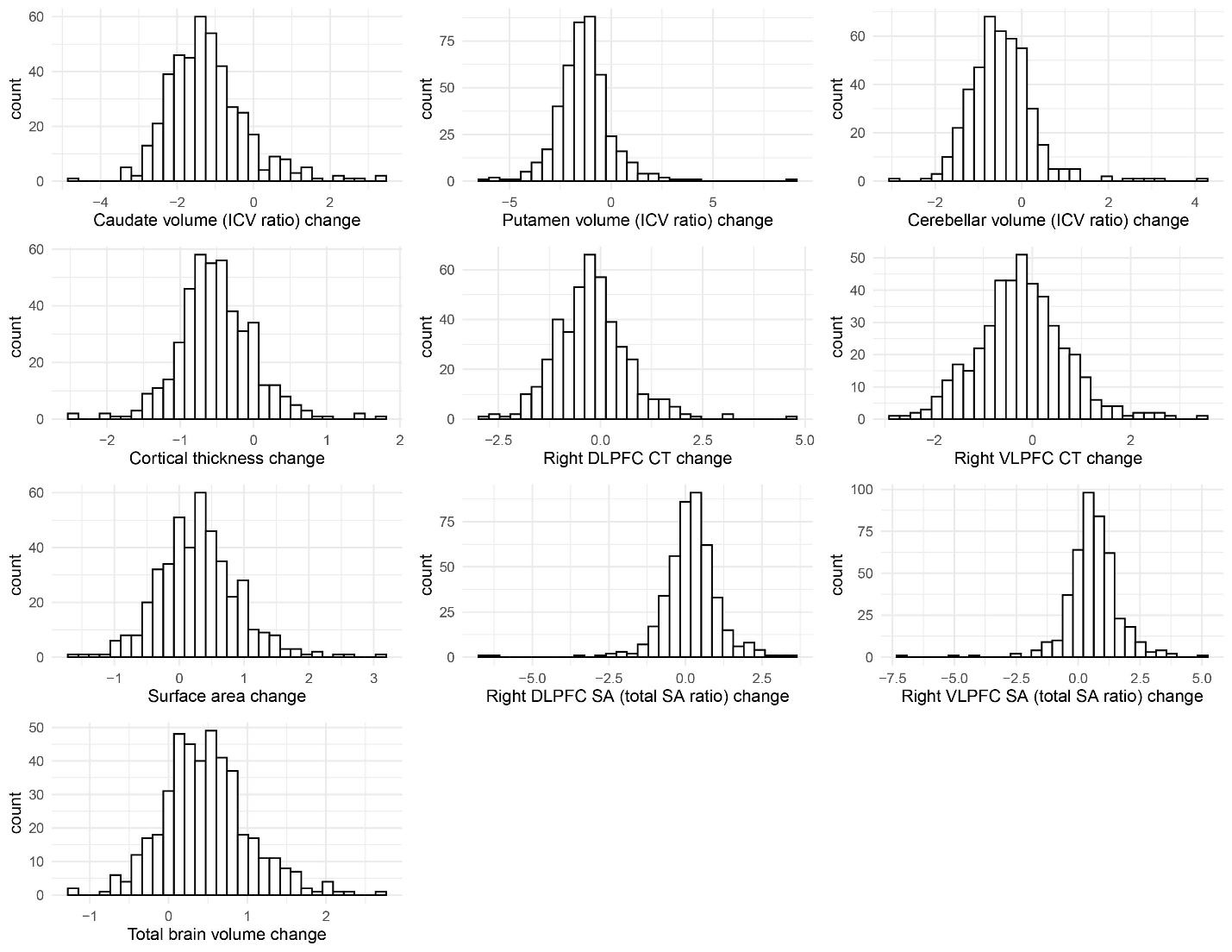


# Figure S14. Distribution of the brain annual change values residualized for the age at baseline scan before normalization in Generation R

CT: cortical thickness; DLPFC: Dorsolateral prefrontal cortex; ICV: intracranial volume; SA: surface area; VLPFC: Ventrolateral prefrontal cortex.


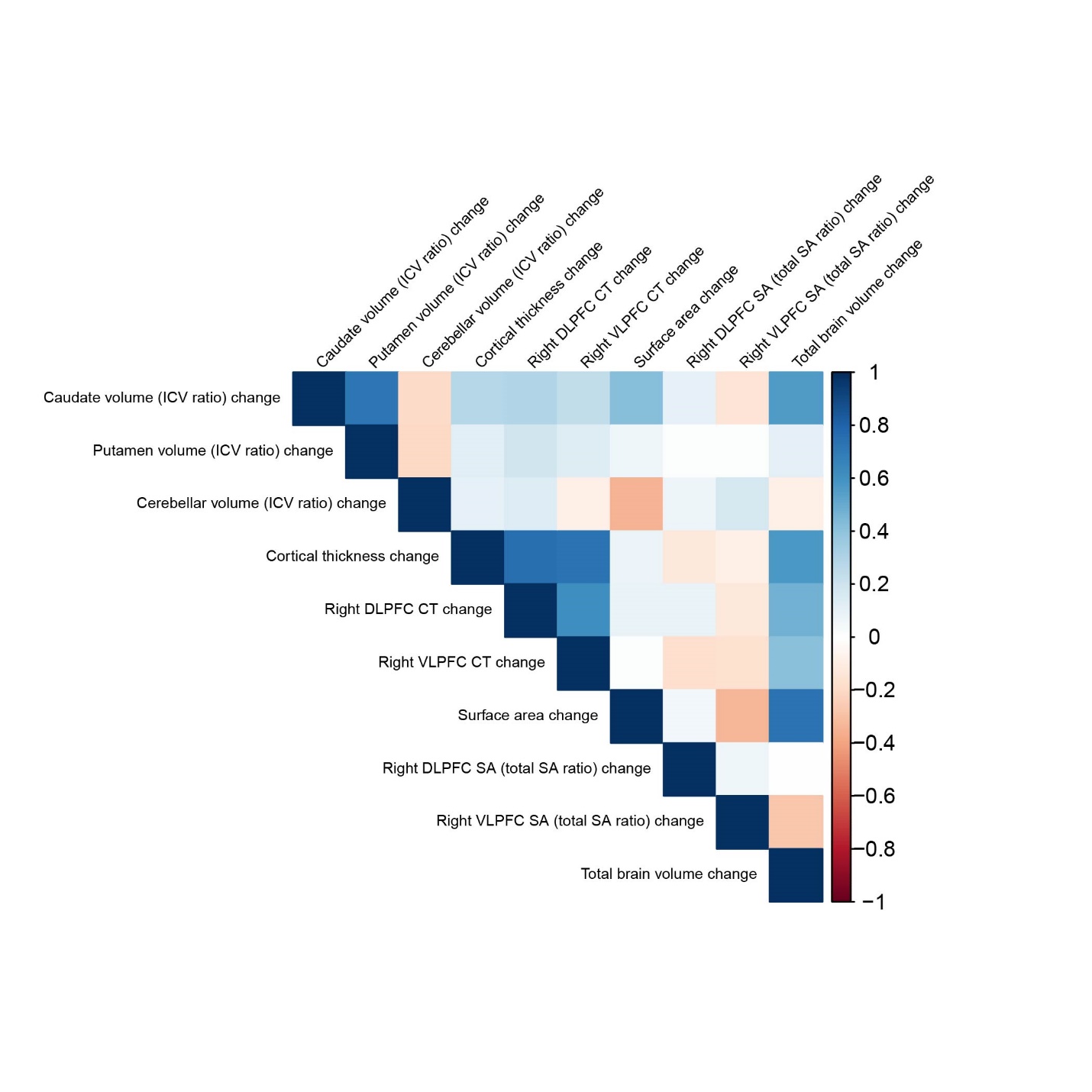


# Figure S15. Correlations among residualized and normalized brain change values in GUSTO

CT: cortical thickness; DLPFC: Dorsolateral prefrontal cortex; ICV: intracranial volume; SA: surface area; VLPFC: Ventrolateral prefrontal cortex.


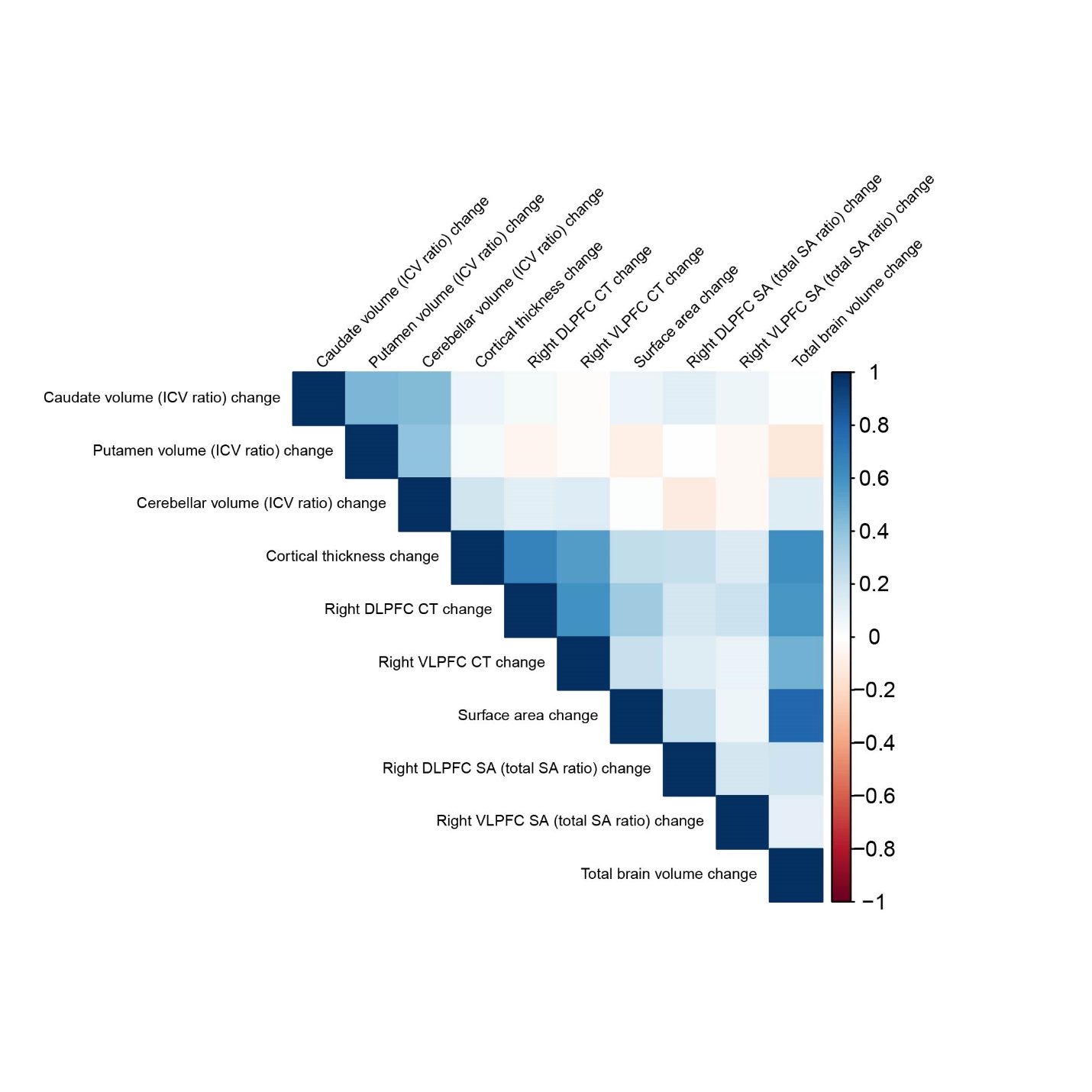


# Figure S16. Correlations among residualized and normalized brain change values in Generation R

CT: cortical thickness; DLPFC: Dorsolateral prefrontal cortex; ICV: intracranial volume; SA: surface area; VLPFC: Ventrolateral prefrontal cortex.

# Table S2. Sex by environment (E) interaction models estimates (separate models for A and M factors)

| **Outcomes^a^** | **A factor** | | | | **M factor** | | | |
| --- | --- | --- | --- | --- | --- | --- | --- | --- |
|  | **Sex** | **E** | **Sex:E** | **R2** | **Sex** | **E** | **Sex:E** | **R2** |
| *GUSTO* | | | | | | | | |
| **Caudate volume^b^ change** | 0.381 | -0.110 | 0.042 | -7.958 | 0.402* | -0.294 | 0.199 | -16.342 |
| **Putamen volume^b^ change** | 0.185 | 0.044 | -0.062 | -0.381 | 0.206 | -0.033 | -0.085 | -0.120 |
| **Cerebellar volume^b^ change** | -0.116 | 0.107 | -0.120 | -0.767 | -0.094 | -0.047 | 0.133 | -1.461 |
| **Cortical thickness change** | 0.165 | 0.048 | -0.177 | -6.044 | 0.175 | -0.167 | 0.233 | -7.691 |
| **Right DLPFC CT change** | 0.196 | 0.069 | -0.225 | -0.704 | 0.197 | -0.065 | 0.033 | -1.716 |
| **Right VLPFC CT change** | 0.165 | 0.171 | -0.215 | -0.092 | 0.203 | -0.072 | 0.108 | -0.143 |
| **Surface area change** | 0.433* | -0.093 | -0.100 | -5.617 | 0.409* | -0.151 | 0.103 | -11.013 |
| **Right DLPFC SA^c^ change** | -0.022 | 0.049 | 0.066 | -16.434 | 0.023 | -0.119 | 0.193 | -14.031 |
| **Right VLPFC SA^c^ change** | -0.258 | 0.124 | 0.005 | -3.349 | -0.253 | 0.221 | -0.134 | -0.714 |
| **Total brain volume change** | 0.454* | 0.003 | -0.171 | -14.477 | 0.443* | -0.123 | 0.158 | -17.030 |
| *Generation R* | | | | | | | | |
| **Caudate volume^b^ change** | -0.039 | -0.180** | 0.085 | -0.011 | -0.039 | -0.002 | -0.022 | -0.030 |
| **Putamen volume^b^ change** | -0.132 | -0.206** | 0.127 | 0.000 | -0.134 | -0.069 | -0.022 | -0.015 |
| **Cerebellar volume^b^ change** | -0.231* | -0.098 | 0.024 | -0.044 | -0.231* | -0.007 | 0.102 | -0.042 |
| **Cortical thickness change** | -0.314** | -0.074 | 0.078 | -0.018 | -0.314** | -0.033 | 0.097 | -0.006 |
| **Right DLPFC CT change** | -0.165 | -0.081 | 0.087 | -0.022 | -0.164 | 0.069 | -0.030 | -0.022 |
| **Right VLPFC CT change** | -0.079 | -0.059 | 0.106 | -0.029 | -0.077 | 0.035 | 0.062 | -0.024 |
| **Surface area change** | -0.337*** | -0.008 | -0.028 | 0.008 | -0.337*** | 0.038 | -0.023 | 0.011 |
| **Right DLPFC SA^c^ change** | 0.008 | -0.067 | 0.126 | -0.030 | 0.009 | -0.020 | 0.126 | -0.024 |
| **Right VLPFC SA^c^ change** | 0.180 | -0.089 | -0.001 | -0.015 | 0.180 | 0.076 | -0.092 | -0.035 |
| **Total brain volume change** | -0.436*** | -0.024 | 0.053 | 0.027 | -0.435*** | 0.031 | 0.040 | 0.036 |

^a^Annual rates of change residualized on age at baseline scan and normalized using rank-based inverse normal transformation. ^b^Adjusted for intracranial volume (ratios). ^c^Adjusted for total surface area (ratios). DLPFC: Dorsolateral prefrontal cortex; VLPFC: Ventrolateral prefrontal cortex; CT: cortical thickness; SA: surface area. Reference=boys. Models adjusted for the first three (GUSTO) or four (Generation R) principal components from GWAS principal component analysis to account for genetic ancestry. ‘***’ 0.001 ‘**’ 0.01 ‘*’ 0.05.

# Table S3. Gene (G, ADHD PGS) by environment (E) interaction models estimates (separate models for A and M factors)

| **Outcomes^a^** | **ADHD PGS threshold** | **A factor** | | | | **M factor** | | | | |
| --- | --- | --- | --- | --- | --- | --- | --- | --- | --- | --- |
|  |  | **G** | **E** | **G:E** | **R2** | **G** | **E** | **G:E** | **R2** |  |
| *GUSTO* | | | | | | | | | | |
| **Caudate volume^b^ change** | 0.05 | -0.139 | -0.094 | 0.162 | -4.869 | -0.149 | -0.153 | -0.009 | -1.661 |  |
| **Putamen volume^b^ change** | 0.001 | -0.159 | 0.041 | -0.069 | -0.263 | -0.138 | -0.095 | 0.138 | -0.131 |  |
| **Cerebellar volume^b^ change** | 0.2 | 0.016 | 0.053 | -0.077 | -0.454 | -0.005 | 0.044 | 0.131 | -1.160 |  |
| **Cortical thickness change** | 0.1 | 0.085 | -0.074 | 0.098 | -3.203 | 0.086 | -0.019 | -0.062 | -7.309 |  |
| **Right DLPFC CT change** | 0.001 | 0.155 | -0.111 | 0.232* | -0.144 | 0.112 | -0.052 | 0.002 | -1.674 |  |
| **Right VLPFC CT change** | 1.00E-05 | -0.058 | 0.044 | 0.127 | -0.165 | -0.035 | -0.025 | -0.056 | -0.118 |  |
| **Surface area change** | 1.00E-08 | 0.099 | -0.159 | -0.071 | -4.240 | 0.071 | -0.067 | 0.069 | -1.045 |  |
| **Right DLPFC SA^c^ change** | 0.001 | 0.069 | 0.046 | 0.197 | -1.121 | 0.047 | 0.002 | 0.024 | -1.336 |  |
| **Right VLPFC SA^c^ change** | 0.0001 | 0.102 | 0.122 | -0.055 | -3.487 | 0.094 | 0.156 | 0.106 | -0.396 |  |
| **Total brain volume change** | 0.05 | 0.076 | -0.109 | 0.048 | -1.106 | 0.080 | -0.024 | -0.119 | -1.657 |  |
| *Generation R* | | | | | | | | | | |
| **Caudate volume^b^ change** | 0.2 | -0.038 | -0.143** | 0.080 | -0.007 | -0.065 | -0.054 | 0.066 | -0.022 |  |
| **Putamen volume^b^ change** | 1.00E-06 | 0.065 | -0.148** | -0.014 | 0.006 | 0.071 | -0.055 | -0.049 | -0.003 |  |
| **Cerebellar volume^b^ change** | 0.2 | -0.115* | -0.068 | -0.015 | -0.034 | -0.133** | 0.059 | 0.011 | -0.030 |  |
| **Cortical thickness change** | 1.00E-07 | -0.044 | -0.043 | **0.174***** | 0.005 | -0.044 | 0.017 | 0.022 | -0.015 |  |
| **Right DLPFC CT change** | 1.00E-07 | -0.047 | -0.042 | 0.083 | -0.015 | -0.051 | 0.072 | -0.038 | -0.013 |  |
| **Right VLPFC CT change** | 1.00E-05 | 0.085 | -0.031 | 0.115* | -0.031 | 0.070 | 0.043 | 0.036 | -0.016 |  |
| **Surface area change** | 1.00E-06 | 0.078 | -0.026 | -0.003 | 0.009 | 0.076 | 0.036 | -0.029 | 0.015 |  |
| **Right DLPFC SA^c^ change** | 1.00E-06 | 0.072 | -0.009 | -0.027 | -0.020 | 0.069 | 0.047 | -0.003 | -0.023 |  |
| **Right VLPFC SA^c^ change** | 0.05 | -0.047 | -0.096* | 0.122** | 0.006 | -0.066 | -0.003 | 0.056 | -0.021 |  |
| **Total brain volume change** | 0.0001 | 0.091 | -0.021 | 0.087 | 0.033 | 0.082 | 0.044 | 0.004 | 0.035 |  |

^a^Annual rates of change residualized on age at baseline scan and normalized using rank-based inverse normal transformation. ^b^Adjusted for intracranial volume (ratios). ^c^Adjusted for total surface area (ratios). DLPFC: Dorsolateral prefrontal cortex; VLPFC: Ventrolateral prefrontal cortex; CT: cortical thickness; SA: surface area. Models adjusted for sex and the first three (GUSTO) or four (Generation R) principal components from GWAS principal component analysis to account for genetic ancestry. ‘***’ 0.001 ‘**’ 0.01 ‘*’ 0.05. **p < 0.006** (Threshold for effective number of tests).

# Table S4. Gene (G, Major Depression and Schizophrenia PGS) by environment (E, A and M factors) interaction models estimates

| **Outcomes^a^** | **PGS threshold** | **G** | **E** | **G:E** | **Specific E contributions** | | **R2** |
| --- | --- | --- | --- | --- | --- | --- | --- |
|  |  |  |  |  | **M factor** | **A factor** |  |
| **Major Depression** | | | | | | | |
| *GUSTO* | | | | | | | |
| **Right DLPFC CT change** | 1.00E-08 | 0.131 | -0.094 | 0.022 | 0.467 | 0.533 | -0.335 |
| *Generation R* | | | | | | | |
| **Caudate volume^b^ change** | 0.001 | -0.036 | -0.162** | 0.135** | 0.123 | 0.877*** | 0.010 |
| **Cerebellar volume^b^ change** | 0.2 | 0.049 | -0.225** | 0.034 | -0.437 | 0.563* | -0.043 |
| **Cortical thickness change** | 0.05 | -0.026 | -0.053 | 0.092* | 0.821 | 0.179 | -0.009 |
| **Right DLPFC CT change** | 0.3 | -0.082 | 0.111 | 0.092 | 0.553* | -0.447 | -0.007 |
| **Right VLPFC CT change** | 0.1 | -0.026 | 0.091 | 0.064 | 0.639* | -0.361 | -0.026 |
| **Right VLPFC SA^c^ change** | 0.001 | -0.063 | -0.192* | 0.023 | -0.396 | 0.604* | -0.015 |
| **Schizophrenia** | | | | | | | |
| *Generation R* | | | | | | | |
| **Caudate volume^b^ change** | 0.4 | -0.042 | -0.178** | 0.163*** | 0.307 | 0.693*** | 0.007 |
| **Cerebellar volume^b^ change** | 1.00E-07 | 0.034 | -0.214* | 0.042 | -0.439 | 0.561* | -0.041 |
| **Cortical thickness change** | 0.5 | -0.046 | 0.095 | -0.068 | 0.669 | -0.331 | -0.021 |
| **Right DLPFC CT change** | 1.00E-05 | 0.078 | 0.134 | -0.043 | 0.573 | -0.427 | -0.018 |
| **Right VLPFC CT change** | 0.4 | -0.038 | 0.154* | -0.096 | 0.725* | -0.275 | -0.028 |
| **Right VLPFC SA^c^ change** | 0.2 | 0.030 | -0.144* | 0.080 | -0.189 | 0.811** | -0.012 |

^a^Annual rates of change residualized on age at baseline scan and normalized using rank-based inverse normal transformation. ^b^Adjusted for intracranial volume (ratios).  ^c^Adjusted for total surface area (ratios). DLPFC: Dorsolateral prefrontal cortex; VLPFC: Ventrolateral prefrontal cortex; CT: cortical thickness; SA: surface area. Models adjusted for sex and the first three (GUSTO) or four (Generation R) principal components from GWAS principal component analysis to account for genetic ancestry. ‘***’ 0.001 ‘**’ 0.01 ‘*’ 0.05.

| **Outcomes^a^** | **ADHD PGS threshold** | **G** | **E** | **G:E** | **Specific E contributions** | | **R2** |
| --- | --- | --- | --- | --- | --- | --- | --- |
|  |  |  |  |  | **M factor** | **A factor** |  |
| **Caudate volume^b^ change** | 0.05 | -0.038 | **-0.180**** | 0.095 | 0.127 | 0.873** | -0.012 |
| **Putamen volume^b^ change** | 1.00E-06 | 0.083 | **-0.183**** | -0.037 | 0.393 | 0.607* | -0.015 |
| **Cerebellar volume^b^ change** | 0.4 | -0.129** | 0.235* | 0.032 | 0.576** | -0.424 | -0.017 |
| **Cortical thickness change** | 1.00E-07 | -0.045 | -0.087 | **0.218***** | -0.168 | 0.832*** | 0.005 |
| **Right DLPFC CT change** | 0.2 | -0.010 | -0.063 | **0.163**** | -0.092 | 0.908** | -0.008 |
| **Right VLPFC CT change** | 0.1 | 0.026 | 0.125 | 0.015 | 0.761 | -0.239 | -0.028 |
| **Surface area change** | 1.00E-06 | 0.083 | 0.079 | -0.057 | 0.611 | -0.389 | 0.001 |
| **Right DLPFC SA^c^ change** | 0.0001 | 0.002 | 0.097 | 0.015 | 0.714 | -0.286 | -0.062 |
| **Right VLPFC SA^c^ change** | 0.05 | -0.048 | -0.107* | 0.116* | 0.006 | 0.994** | 0.008 |
| **Total brain volume change** | 1.00E-07 | 0.020 | -0.028 | 0.132* | -0.130 | 0.870* | -0.003 |

# Table S5. Gene (G, ADHD PGS) by environment (E, A and M factors) interaction models estimates excluding children taking ADHD medication (n=17) in Generation R

^a^Annual rates of change residualized on age at baseline scan and normalized using rank-based inverse normal transformation. ^b^Adjusted for intracranial volume (ratios). ^c^Adjusted for total surface area (ratios). DLPFC: Dorsolateral prefrontal cortex; VLPFC: Ventrolateral prefrontal cortex; CT: cortical thickness; SA: surface area. Models adjusted for sex and the first four principal components from GWAS principal component analysis to account for genetic ancestry. ‘***’ 0.001 ‘**’ 0.01 ‘*’ 0.05. **p < 0.006** (Threshold for effective number of tests).

# Table S6. Gene (G, ADHD PGS) by environment (E, A and M factors) by sex interaction models estimates

| **Outcomes^a^** | **ADHD PGS threshold** | **G** | **E** | **Sex** | **G:E:Sex** | **Specific E contributions** | | **R2** |
| --- | --- | --- | --- | --- | --- | --- | --- | --- |
|  |  |  |  |  |  | **M factor** | **A factor** |  |
| *GUSTO* | | | | | | | | |
| **Caudate volume^b^ change** | 1.00E-06 | -0.070 | -0.055 | 0.385* | -0.590* | -0.256 | 0.744** | -6.195 |
| **Putamen volume^b^ change** | 1.00E-07 | 0.158 | -0.219 | 0.194 | -0.695 | 0.546* | -0.454* | -0.117 |
| **Cerebellar volume^b^ change** | 0.2 | -0.028 | -0.235 | -0.060 | 0.880* | 0.609** | -0.391 | -0.115 |
| **Cortical thickness change** | 0.05 | 0.193 | -0.269 | 0.148 | -0.383 | 0.486* | -0.514* | -2.943 |
| **Right DLPFC CT change** | 0.001 | 0.204 | 0.030 | 0.142 | 0.234 | 0.196 | 0.804** | -0.150 |
| **Right VLPFC CT change** | 1.00E-05 | -0.124 | 0.403 | 0.140 | 0.062 | -0.378 | 0.622* | -0.088 |
| **Surface area change** | 0.3 | 0.142 | 0.048 | 0.332 | -0.784* | 0.432* | -0.568** | -1.111 |
| **Right DLPFC SA^c^ change** | 0.1 | 0.062 | -0.061 | -0.022 | 0.115 | 0.344 | 0.656* | -9.586 |
| **Right VLPFC SA^c^ change** | 5.00E-08 | 0.006 | 0.310 | -0.271 | -0.200 | 0.643 | 0.357 | -1.036 |
| **Total brain volume change** | 0.05 | 0.117 | 0.113 | 0.395* | 0.834* | -0.413* | 0.587** | -6.702 |
| *Generation R* | | | | | | | | |
| **Caudate volume^b^ change** | 1.00E-06 | -0.077 | **-0.330**** | -0.037 | -0.038 | -0.398 | 0.602*** | -0.013 |
| **Putamen volume^b^ change** | 0.05 | -0.188** | -0.194** | -0.128 | 0.008 | 0.076 | 0.924** | -0.004 |
| **Cerebellar volume^b^ change** | 0.05 | **-0.260***** | 0.151 | -0.217* | -0.202 | 0.468* | -0.532* | -0.027 |
| **Cortical thickness change** | 1.00E-08 | -0.130 | -0.094 | **-0.321***** | 0.116 | 0.071 | 0.929** | 0.001 |
| **Right DLPFC CT change** | 0.2 | -0.082 | -0.112 | -0.194* | 0.229 | -0.158 | 0.842*** | -0.019 |
| **Right VLPFC CT change** | 1.00E-05 | 0.048 | -0.090 | -0.073 | -0.069 | 0.146 | 0.854* | -0.036 |
| **Surface area change** | 0.5 | 0.067 | -0.058 | **-0.359***** | 0.324* | -0.357 | 0.643* | 0.007 |
| **Right DLPFC SA^c^ change** | 1.00E-08 | 0.063 | 0.013 | 0.003 | 0.025 | 0.745 | 0.255 | -0.032 |
| **Right VLPFC SA^c^ change** | 0.05 | 0.001 | -0.088 | 0.168 | 0.079 | 0.026 | 0.974** | -0.012 |
| **Total brain volume change** | 0.3 | 0.117 | -0.054 | **-0.466***** | 0.196 | -0.141 | 0.859 | 0.022 |

^a^Annual rates of change residualized on age at baseline scan and normalized using rank-based inverse normal transformation. ^b^Adjusted for intracranial volume (ratios). ^c^Adjusted for total surface area (ratios). DLPFC: Dorsolateral prefrontal cortex; VLPFC: Ventrolateral prefrontal cortex; CT: cortical thickness; SA: surface area. Reference=boys. Models adjusted for the first three (GUSTO) or four (Generation R) principal components from GWAS principal component analysis to account for genetic ancestry. ‘***’ 0.001 ‘**’ 0.01 ‘*’ 0.05. **p < 0.006** (Threshold for effective number of tests).

**References**

1. Cecil CA, Lysenko LJ, Jaffee SR, et al. Environmental risk, Oxytocin Receptor Gene (OXTR) methylation and youth callous-unemotional traits: a 13-year longitudinal study. Mol Psychiatry. Oct 2014;19(10):1071-7.

2. Rijlaarsdam J, Pappa I, Walton E, et al. An epigenome-wide association meta-analysis of prenatal maternal stress in neonates: A model approach for replication. Epigenetics. 2016;11(2):140-9.

3. Rosseel Y. lavaan: An R Package for Structural Equation Modeling. Journal of Statistical Software. 2012;48(2):1 - 36. doi:10.18637/jss.v048.i02

4. van Buuren S, Groothuis-Oudshoorn K. mice: Multivariate Imputation by Chained Equations in R. Journal of Statistical Software. 2011;45(3):1 - 67. doi:10.18637/jss.v045.i03

5. Szekely E, Neumann A, Sallis H, et al. Maternal Prenatal Mood, Pregnancy-Specific Worries, and Early Child Psychopathology: Findings From the DREAM BIG Consortium. J Am Acad Child Adolesc Psychiatry. Jan 2021;60(1):186-197.

6. Reichenheim ME, Moraes CL, Oliveira AS, Lobato G. Revisiting the dimensional structure of the Edinburgh Postnatal Depression Scale (EPDS): empirical evidence for a general factor. BMC Med Res Methodol. Jun 20 2011;11:93.

7. Lin X, Teh AL, Chen L, et al. Choice of surrogate tissue influences neonatal EWAS findings. BMC Med. Dec 5 2017;15(1):211.

8. Medina-Gomez C, Felix JF, Estrada K, et al. Challenges in conducting genome-wide association studies in highly admixed multi-ethnic populations: the Generation R Study. Eur J Epidemiol. Apr 2015;30(4):317-30.

9. Wen DJ, Poh JS, Ni SN, et al. Influences of prenatal and postnatal maternal depression on amygdala volume and microstructure in young children. Transl Psychiatry. Apr 25 2017;7(4):e1103.

10. White T, Muetzel RL, El Marroun H, et al. Paediatric population neuroimaging and the Generation R Study: the second wave. Eur J Epidemiol. Jan 2018;33(1):99-125.

11. Jansen PR, Dremmen M, van den Berg A, et al. Incidental Findings on Brain Imaging in the General Pediatric Population. N Engl J Med. Oct 19 2017;377(16):1593-1595.
